# Adipose-enriched peri-tumoral stroma, in contrast to myofibroblast-enriched stroma, prognosticates poorer survival in breast cancers

**DOI:** 10.1038/s41523-023-00590-7

**Published:** 2023-10-20

**Authors:** Hannah Si Hui Lau, Veronique Kiak Mien Tan, Benita Kiat Tee Tan, Yirong Sim, Jelmar Quist, Aye Aye Thike, Puay Hoon Tan, Shazib Pervaiz, Anita Grigoriadis, Kanaga Sabapathy

**Affiliations:** 1 Divisions of Cellular & Molecular Research, National Cancer Centre Singapore, Singapore, 168583 Singapore; 2 Department of Physiology, Yong Loo Lin School of Medicine, National University of Singapore, Singapore, 119228 Singapore; 3 Cancer Bioinformatics, School of Cancer & Pharmaceutical Sciences, Faculty of Life Sciences and Medicine, King’s College London, London, UK; 4 Surgery and Surgical Oncology, National Cancer Centre Singapore, Singapore, 169610 Singapore; 5 Department of Breast Surgery, Singapore General Hospital, Singapore, 168753 Singapore; 6 Department of General Surgery, Sengkang General Hospital, Singapore, 544886 Singapore; 7 Breast Cancer Now Research Unit, School of Cancer and Pharmaceutical Sciences, Faculty of Life Sciences and Medicine, King’s College London, London, UK; 8 Division of Pathology, Singapore General Hospital, Singapore, 169856 Singapore; 9 NUS Centre for Cancer Research (N2CR), Yong Loo Lin School of Medicine, National University of Singapore, Singapore, 119228 Singapore; 10 School of Biological Sciences, Nanyang Technological University, Singapore, 637551 Singapore

**Keywords:** Breast cancer, Tumour heterogeneity

## Abstract

Despite our understanding of the genetic basis of intra-tumoral heterogeneity, the role of stromal heterogeneity arising from an altered tumor microenvironment in affecting tumorigenesis is poorly understood. In particular, extensive study on the peri-tumoral stroma in the morphologically normal tissues surrounding the tumor is lacking. Here, we examine the heterogeneity in tumors and peri-tumoral stroma from 8 ER^+^/PR^+^/HER2^−^ invasive breast carcinomas, through multi-region transcriptomic profiling by microarray. We describe the regional heterogeneity observed at the intrinsic molecular subtype, pathway enrichment, and cell type composition levels within each tumor and its peri-tumoral region, up to 7 cm from the tumor margins. Moreover, we identify a pro-inflammatory adipose-enriched peri-tumoral subtype which was significantly associated with poorer overall survival in breast cancer patients, in contrast to an adaptive immune cell- and myofibroblast-enriched subtype. These data together suggest that peri-tumoral heterogeneity may be an important determinant of the evolution and treatment of breast cancers.

## Introduction

Breast cancers are highly heterogenous, displaying a great degree of diversity within and between them^1^. While intra-tumoral heterogeneity (ITH) has been well studied from the perspective of tumor-cell autonomous genetic variations^2–5^, the functional interactions between the tumor and non-malignant stromal and immune cells of the tumor microenvironment (TME) in dictating ITH are not fully understood^6^. Moreover, most studies on the TME have focused on using intra-tumoral stroma located within the tumor mass, with a stark deficiency in studies examining the peri-tumoral microenvironment, which extends further beyond the tumor margins and includes the surrounding histologically normal, benign-appearing tissues^7^.

Although histologically normal tissues adjacent to the tumor are generally used as healthy controls in cancer studies, one study comparing the transcriptomic profiles of these tissues (>2 cm from the tumor margin) from The Cancer Genome Atlas (TCGA) against healthy tissue from the Genotype-Tissue Expression (GTEx) program across 8 tissue types showed that they represent a distinct, intermediate state between healthy tissue and tumor^8^. This phenomenon is not restricted to human samples as tumor-adjacent normal tissues from mouse models also show an intermediatory transcriptomic profile^9^.

Among breast cancer patients, transcriptomic profiles of tumor-adjacent normal tissues compared to reduction mammoplasty tissues showed alterations in wound healing response^10^, lipid metabolism, epithelial-mesenchymal transition (EMT), and extracellular matrix (ECM) organization^11^, suggesting that the tumor is likely to impose its influences on the surrounding tissue over a substantial distance.

Given that different regions within a tumor mass have been shown to possess distinct molecular features, we wanted to explore whether this impacts the adjacent peri-tumoral regions accordingly to diversify the stromal composition. Moreover, the existence of gradient effects from the tumor mass on stromal composition has also not been well explored. As such, we embarked on a systematic multi-region transcriptomic profiling analysis of the tumor and morphologically normal, peri-tumoral tissues in 8 ER^+^/PR^+^/HER2^−^ breast tumors.

Using multi-region sampling, we characterize the heterogeneity within breast tumors and peri-tumoral samples collected from different sides and distances surrounding the tumor mass at the molecular subtype, pathway enrichment, and cell type composition levels. Our analyses on the peri-tumoral samples reveal four clusters that differ in molecular signaling pathway activities and cell type composition but are independent of distance and direction from the tumor. Furthermore, these peri-tumoral gene expression signatures demonstrate prognostic value in predicting patient survival outcomes in publicly available data sets. Our findings thus suggest that different pre-existing resident stromal cells in the peri-tumoral regions are influenced by the tumor differently and may in turn have varying effects on tumor growth and development.

## Results

### Cohort characteristics

**Fig. 1:**
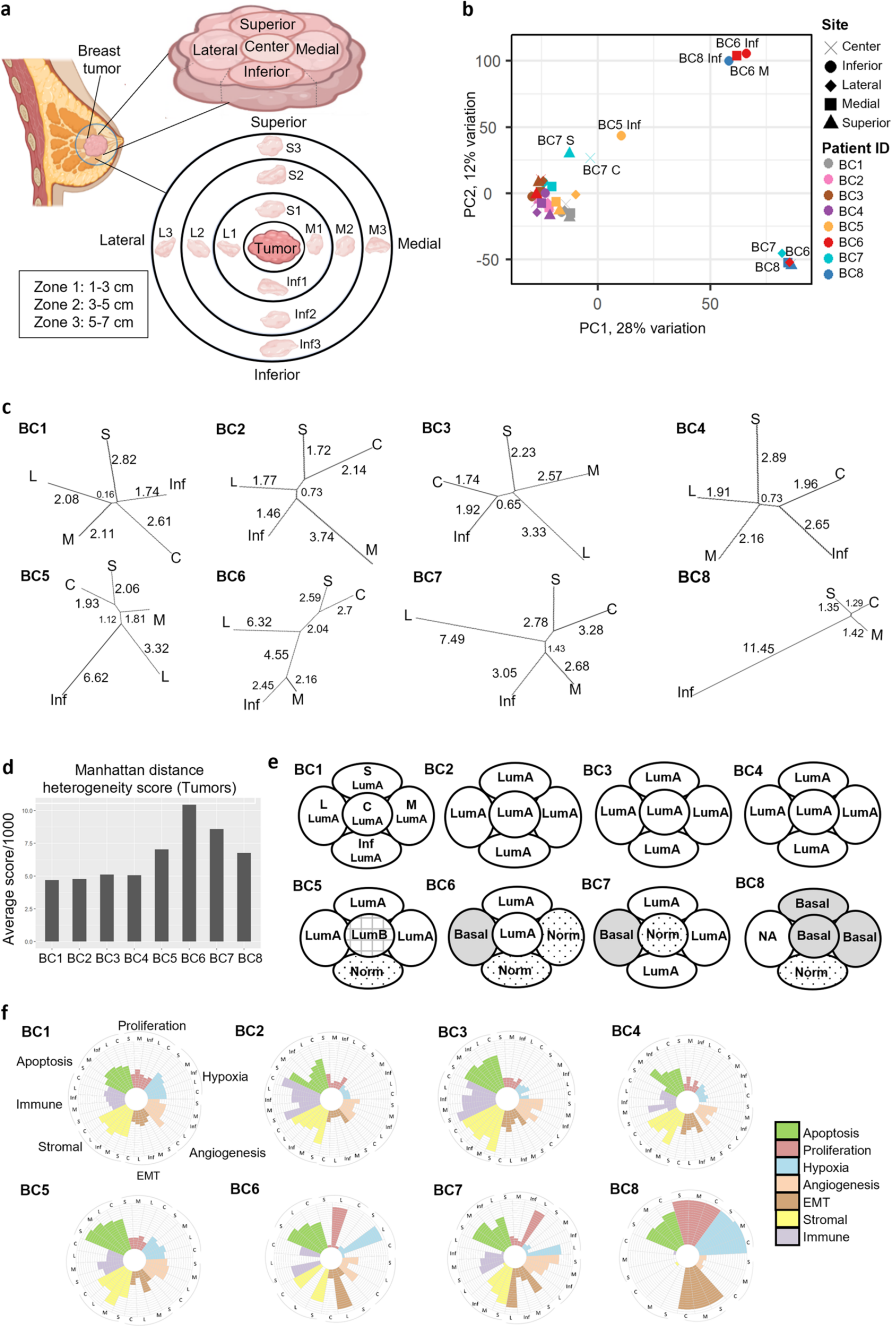
Tumor samples from ER^+^/PR^+^/HER2^−^ patients show inter-tumor and intra-tumor heterogeneity. **a** Schematic representation of the study design showing multi-regional profiling of tumor (S Superior, M Medial, Inf Inferior, L Lateral, C Center) and peri-tumoral stroma in breast cancer patients. **b** Principal component analysis (PCA) plot showing the extent of transcriptomic variation across tumor samples. Patient ID (e.g., BC1) is indicated by color while the tumor site is indicated by the point shape. **c** Dendrograms representing the divergence in transcriptomic profiles (in terms of branch lengths) between different regions of each patient’s tumor. Dendrograms are plotted using pairwise Manhattan distances calculated using the expression profile of all genes. Values indicate branch lengths (in thousands). **d** Bar plots showing the heterogeneity score for each patient’s tumor, calculated by taking the average pairwise Manhattan distances of all tumor samples for each patient. **e** Spatial diagrams indicate the molecular subtype of each tumor region for each patient (LumA Luminal A, LumB Luminal B, Her2 Her2-enriched, Basal Basal-like, Normal Normal-like, NA Sample not available). **f** Circular bar plots showing scores for key biological processes in tumor samples. Normal-like samples highly enriched for adipocytes/muscle cells have been excluded from analysis.

We investigated the transcriptomic profiles of 8 ER^+^/PR^+^/HER2^−^-classified primary breast tumors from patients who had undergone a mastectomy between 2016 and 2017 at the National Cancer Centre Singapore (Supplementary Dataset 1). Tumor samples were obtained from the center, superior, inferior, medial, and lateral (C, S, Inf, M, L) regions of each tumor mass (Fig. 1a). Peri-tumoral samples were obtained from four radial directions corresponding to the regions the tumor samples were collected from (S, Inf, M, L) and from three zones of increasing distances from the tumor margins (Zone 1: 1–3 cm, Zone 2: 3–5 cm, Zone 3: 5– cm). Due to the location of some tumors, it was not possible to obtain all samples up to 7 cm from the tumor in all directions (e.g., if the tumor was located at a further distance from the nipple). Details of samples collected for each patient are provided in Supplementary Dataset 1. To represent the normal breast transcriptomic state, 5 non-directional samples were each obtained from 3 non-tumor bearing breasts (NTB)—1 patient had undergone cosmetic reduction mammoplasty and 2 patients were germline *BRCA2-*mutation carriers who had undergone risk-reducing mastectomies. Total RNA was isolated and microarray was performed using the human Clariom™ S Assay (ThermoFisher Scientific). In total, 126 transcriptomic profiles (39 tumor, 72 peri-tumoral, 15 NTB) were analyzed.

### Multiple molecular subtypes within ER^+^PR^+^HER2^−^ breast tumors contribute to ITH

We first evaluated the overall transcriptomic heterogeneity among the tumor samples by performing principal component analysis (PCA) on all 39 tumor samples which revealed 1 large cluster with over 70% (*n* = 28) of samples and 3 smaller clusters consisting of 3 to 5 samples each (Fig. 1b). While 4 patients had all tumor samples clustered together (BC1-4), the remaining 4 patients had samples in multiple PCA clusters (Supplementary Fig. 1a), suggesting varying levels of ITH between patients. This was further validated by constructing dendrograms such that branch lengths indicate the extent of transcriptomic divergence between each region (Fig. 1c), identifying which samples have the most divergent transcriptomic profiles compared to other samples within the same patient. A Manhattan distance-based summary ITH score for each patient (Fig. 1d) identified patients BC5, 6, 7 and 8 as harboring the most heterogeneous tumors.

We next tested whether ITH is also reflected in their molecular breast cancer subtype classification. As expected, for the ER^+^/PR^+^/HER2^−^ cohort, the Absolute Intrinsic Molecular Subtyping (AIMS)^12^ and PAM50^13^ classifiers confirmed that 29 out of 39 samples were Luminal A/B (Fig. 1e), while the remaining samples were classified as either Normal-like or Basal-like. The Normal-like tumor samples from patients BC5 to 8 were further separated into 2 clusters in the PCA (Supplementary Fig. 1b), and found to be either enriched for adipose tissue (BC5-Inf and BC7-C) based on pathway enrichment analysis of significantly upregulated genes (logFC>1, adj. *p* value < 0.05) (Supplementary Fig. 1c), or enriched for myocytes and skeletal muscle cells (BC6-M, BC6-Inf and BC8-Inf), as inferred by xCell cell type enrichment analysis^14^ and pathway enrichment analysis (Supplementary Fig. 1d, e, Supplementary Dataset 2). Muscle-like features were seen in respective hematoxylin and eosin (H&E) images (Supplementary Fig. 1f), which may potentially be derived from the pectoral muscles underlying the breast. As such, these non-epithelial enriched samples were excluded in subsequent analyses.

Three tumors from patients BC6, 7 and 8 contained basal-classified samples (Fig. 1e), despite patients being classified histologically as ER^+^/PR^+^ with ER-positivity above 95% (Supplementary Dataset 1). Pathway enrichment and gene set enrichment analysis (GSEA) showed significant upregulation of genes involved in cell cycle regulation and mitosis (Supplementary Fig. 1g), as well as proliferation-promoting pathways including MYC targets, Wnt/beta-catenin signaling and E2F targets (Supplementary Fig. 1h).

To further understand the ITH of these tumors, we explored various cancer-related pathway activities, including proliferation, apoptosis, hypoxia, angiogenesis and EMT using a Z-scoring approach^15^, and their infiltrating stromal and immune levels by ESTIMATE^16^ (Fig. 1f, Supplementary Dataset 3). ITH at the biological processes level reflected the molecular subtype, as basal-like regions showed significantly higher proliferation, hypoxia and EMT scores, but extremely low immune and stromal infiltration (Supplementary Fig. 1i). Among tumor samples from patients BC1 to 5 which do not have basal-like regions, the immune, stromal, and EMT scores showed the greatest within-patient variation while proliferation and hypoxia scores were generally homogenous among different regions of the same tumor, based on the standard deviation for each patient’s tumor samples. Using CIBERSORTx deconvolution^17^, we investigated regional differences in immune cell type composition within each tumor but this was generally low (Supplementary Fig. 1j). Basal-like samples, however, had significantly lower M1 and M2 macrophage proportions compared to Luminal A/B samples (Mann–Whitney test, *p* < 0.05, Benjamini–Hochberg adjustment).

These results together demonstrate that even in tumors identified as ER^+^/PR^+^/HER^−^ by histological classification, some tumors display gene expression patterns reflecting multiple molecular subtypes within a single tumor, showing significant transcriptomic and phenotypic ITH.

### Landscape of peri-tumoral transcriptomic heterogeneity

**Fig. 2:**
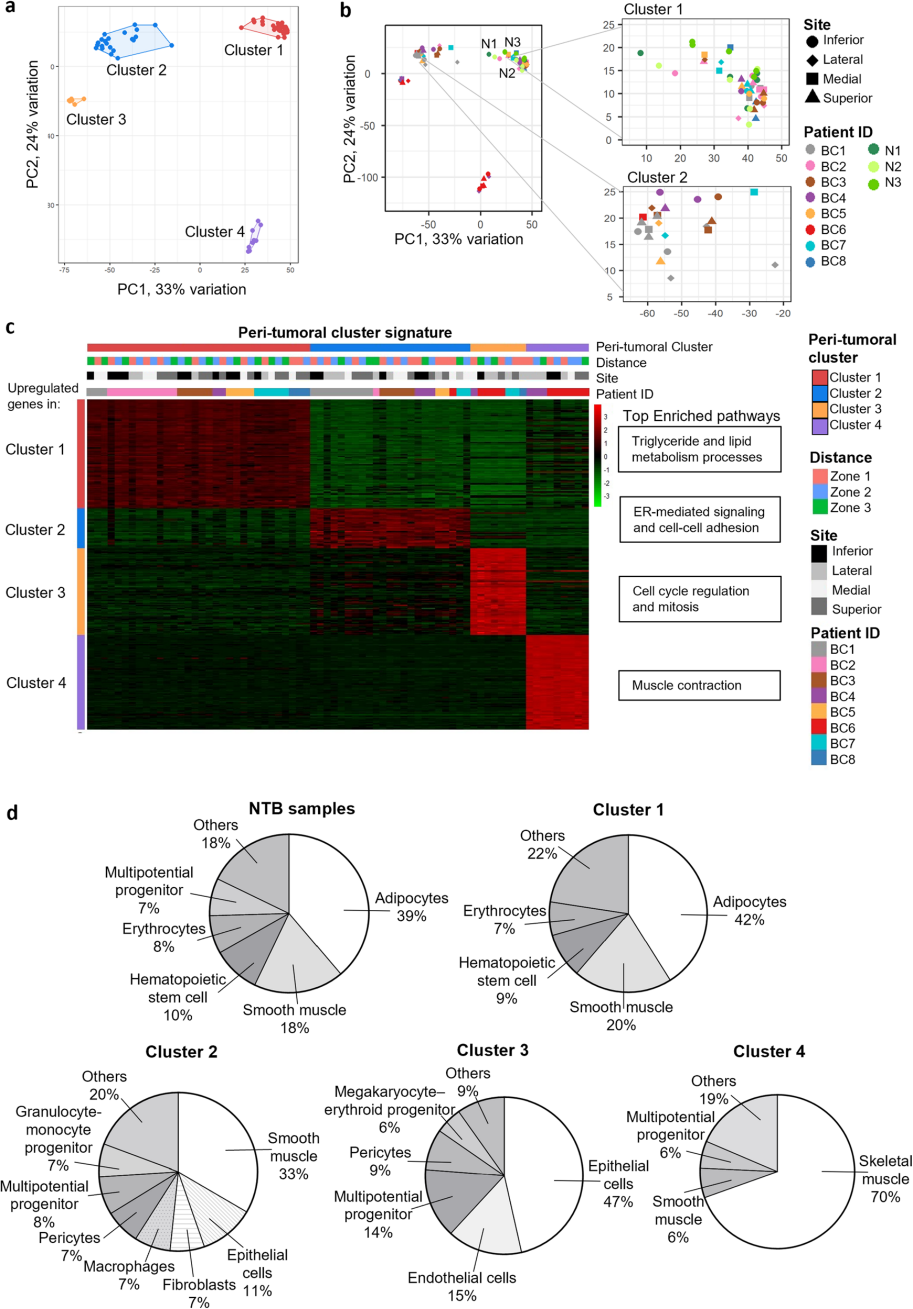
Four clusters are identified from morphologically normal peri-tumoral samples in breast cancer patients. **a** PCA plot showing the extent of transcriptomic variation across all peri-tumoral samples. **b** Left: PCA plot of all peri-tumoral and non-tumor bearing breasts (NTB) samples. Right: Zoomed-in PCA plots for Cluster 1 and 2. Patient ID is indicated by color and samples from NTB are indicated in green (N1 to N3). Site is indicated by the point shape. **c** Heatmap of genes upregulated in peri-tumoral samples from each cluster versus all other peri-tumoral samples (logFC>0.5, adjusted *p* < 0.05). **d** Pie charts showing the average cell type composition of each peri-tumoral cluster by CIBERSORTx deconvolution. Cell types with estimated proportion above 5% are shown.

As these tumors display a range of ITH, they provide an opportunity to explore whether regional heterogeneity within a tumor mass would impact the adjacent peri-tumoral regions accordingly. To understand the overall heterogeneity among the 72 peri-tumoral samples collected, PCA was performed which separated them into four clusters (Fig. 2a) that were validated by K-means clustering and non-negative matrix factorization (Supplementary Fig. 2a, b). We included 15 samples from NTBs which overlapped with the largest cluster, encompassing 32 stroma samples and referred to as Cluster 1 (Fig. 2b). Most of the remaining samples were found in Cluster 2 (*n* = 23), whereas Clusters 3 and 4 consisted of 8 and 9 samples, respectively.

To understand the key transcriptional differences between these 4 clusters, we performed differential gene expression analyses to identify significantly upregulated genes in each cluster compared to all other peri-tumoral samples (logFC>0.5, adjusted *p* value < 0.05) (Fig. 2c, Supplementary Dataset 4), as well as CIBERSORTx using a custom signature gene matrix that allows deconvolution into 28 cell types^18^ (Supplementary Dataset 5). Pathway enrichment analysis on Cluster 1 upregulated genes showed they were largely involved in triglyceride and lipid metabolism processes (Supplementary Fig. 2c) while adipocytes were estimated to make up over 40% of Cluster 1 samples on average (Fig. 2d). The increased presence of adipocytes was confirmed by manual inspection of H&E-stained images (Supplementary Fig. 2d) and GSEA (Supplementary Fig. 2e). Cluster 1 samples also had the most similar cell type composition as NTB samples.

On the other hand, significantly upregulated genes in Cluster 2 were mainly involved in ER-mediated signaling and cell-cell adhesion (Supplementary Fig. 2c, Supplementary Dataset 4). Deconvolution of these samples identified smooth muscle as the most abundant cell type (33.4%), but they also had a significant proportion of epithelial cells (11.2%) and fibroblasts (7%). Increased fibrous stroma proportions was observed in H&E-stained images of these samples (Supplementary Fig. 2d).

Genes upregulated in Cluster 3 were involved in cell cycle regulation and mitosis (Supplementary Fig. 2c) and GSEA further identified enrichment of cell proliferation-promoting pathways such as MYC, Wnt/beta-catenin, and MTORC1 signaling (Supplementary Fig. 2e). CIBERSORTx estimated these samples to be composed of over 40% of epithelial cells on average (Fig. 2d). Given that these samples showed similar phenotypic characteristics as basal-like tumor samples, mainly high proliferation and hypoxia but low stromal and immune scores (Supplementary Fig. 2f), we hypothesize that they may contain infiltrating basal-like tumor cells into the peri-tumoral regions due to their increased invasiveness, despite these samples being morphologically normal.

Lastly, Cluster 4 upregulated genes were involved in muscle related processes and deconvolution estimated that almost 70% was composed of skeletal muscle cells (Fig. 2d). Similar to the muscle-enriched tumor samples, these samples could have been in close proximity to the underlying pectoral muscles. These samples could therefore contain muscle fibers contaminated from the pectoralis muscle tissue during sample collection. As such, our subsequent analyses focus on Clusters 1 and 2 which reflect the major breast stromal states.

Together, these findings suggest that major differences observed in the whole transcriptomes of these peri-tumoral samples are driven by underlying differences in cell type composition.

### Intra-patient regional peri-tumoral heterogeneity

**Fig. 3:**
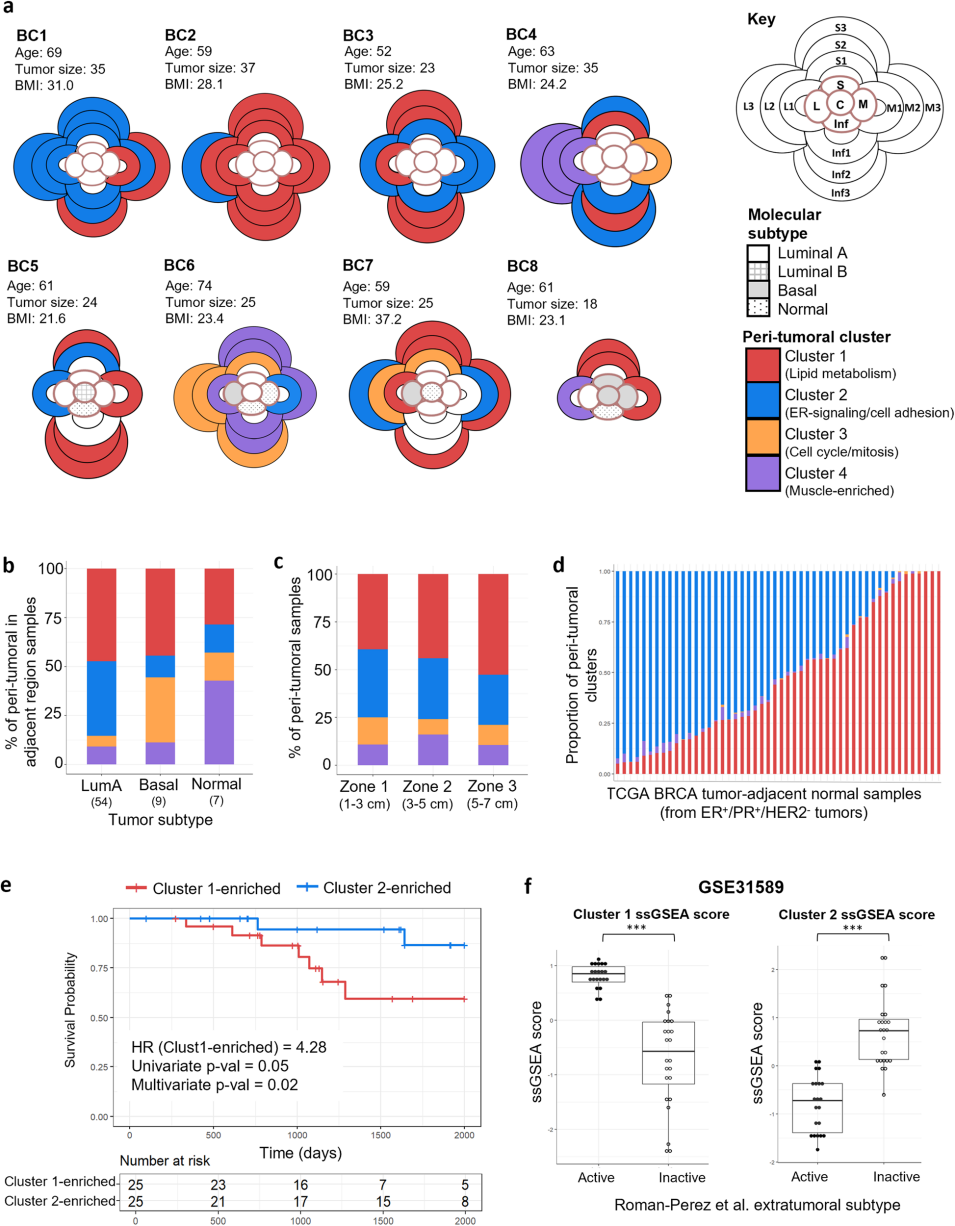
Spatial distribution and prognostic value of peri-tumoral clusters in breast cancer patients. **a** Spatial diagrams indicating the cluster each peri-tumoral sample is assigned to by K-means clustering for each patient. Molecular subtypes of tumor regions are indicated and age of patient, tumor size (mm), and BMI (kg/m^2^) are provided. **b** Bar plots showing the percentage of peri-tumoral samples assigned to each cluster according to the molecular subtype of the adjacent tumor region. Total number of peri-tumoral samples in the corresponding radial direction for each molecular subtype is indicated in brackets. Basal-like tumor samples are significantly associated with Cluster 3 in the adjacent region while normal-like tumor samples are significantly associated with Cluster 4 (Chi-square test, *p* < 0.05). **c** Bar plots showing the percentage of peri-tumoral samples within each zone assigned to each cluster. No significant association between distance zone and cluster assignment was found by chi-square test. **d** Deconvolution analysis of tumor-adjacent normal tissue from TCGA BRCA patients with ER^+^/PR^+^/HER2^−^ tumors (*n* = 50) by CIBERSORTx into the four peri-tumoral clusters. **e** Overall survival curves of TCGA BRCA patients with ER^+^/PR^+^/HER2^−^ tumors (*n* = 50) divided into Cluster 1-enriched and Cluster 2-enriched groups based on CIBERSORTx deconvolution proportions of tumor-adjacent normal tissues. Hazard ratios (HR) and log-rank test *p* values are shown. **f** ssGSEA scores for Cluster 1 and Cluster 2 genes in tumor-adjacent normal samples from ER^+^ patients from GSE31589 (*n* = 45), grouped according to Roman-Perez et al. (2012) annotation for Active/Inactive extratumoral microenvironment subtype. Mann–Whitney *U* test *p* values are shown (***p ≤ 0.001). Center line represents the median, box limits show the upper and lower quartiles and whiskers show 1.5× interquartile ranges.

We next examined how these clusters are distributed among the 8 patients, and spatially within each patient, to understand if they are driven by the biology of the tumor. As shown in the spatial diagrams representing the location each sample was taken from (Fig. 3a), most patients had samples belonging to Clusters 1 and 2 only (BC1-3, BC5), while 4 patients had samples belonging to Cluster 3 as well (BC4, BC6-8) (Supplementary Fig. 3a). Cluster 4 samples were found only in 2 patients (BC4 and BC6). Similar to the ITH analyses, we established a Manhattan distance-based peri-tumoral heterogeneity score for each patient (Supplementary Fig. 3b). When we compared the ITH and peri-tumoral heterogeneity scores, a trend towards a positive correlation was observed (Pearson correlation *r* = 0.518, *p* = 0.153) (Supplementary Fig. 3c).

Within these 8 patients, we did not observe any significant association between age and body mass index (BMI) with the cluster identity of their peri-tumoral samples. However, given that age and obesity are both major risk factors for breast cancer^19,20^ and have been associated with breast density^21,22^, we sought to investigate in a larger cohort if they are also associated with the stromal gene signatures identified from our samples. We obtained RNA-seq data of normal breast samples from 145 healthy women without breast disease^23^ and generated single-sample GSEA (ssGSEA) scores for each sample based on Cluster 1 and 2-upregulated genes found in Fig. 2c. While age was significantly positively associated with the expression of Cluster 1 genes (*p* = 0.002, Supplementary Fig. 3d), no significant association was observed between BMI and Cluster 1 or 2 ssGSEA scores (Supplementary Fig. 3e), suggesting that the adipose-enriched-cluster might not be associated with overall obesity.

We next investigated whether the molecular subtype of the tumor affects the peri-tumoral cluster of the adjacent peri-tumoral samples. LumA tumor samples were not significantly associated with any cluster in the corresponding radial direction but had a large proportion of these peri-tumoral samples assigned to Cluster 1 (*n* = 28) and Cluster 2 (*n* = 21) (Fig. 3b). However, basal-like tumor samples were significantly associated with Cluster 3 in the adjacent region (Chi-square test, *p* < 0.05), supporting our hypothesis that these samples may contain infiltrating basal-like tumor cells into the peri-tumoral regions.

We also investigated whether the above four clusters are located a certain distance from the tumor margins, to determine the existence of any gradient effects from the tumor mass. If present, we hypothesized that peri-tumoral regions most distal would have similar gene expression profiles as the NTB samples due to a weaker tumor field effect. Surprisingly, samples from all four clusters were found randomly in each distance zone (Fig. 3c, Supplementary Fig. 3f). The similarity in overall transcriptomic profiles between Cluster 1 and NTB samples (Fig. 2b) instead appears to be driven by similarity in their cellular composition, with both having a large proportion of adipocytes (Fig. 2d).

Our multi-regional transcriptomic analysis of the surrounding normal tissue in these 8 breast tumors thus demonstrates that the four clusters are mainly driven by variations in breast cellular composition, rather than by the tumor.

### Gene expression in tumor-adjacent normal tissue is associated with overall survival

Having established that each patient has a different peri-tumoral transcriptomic and cellular profile, we next asked if this would prognosticate overall survival in breast cancers. We obtained 50 bulk RNAseq data of tumor-adjacent normal tissues from ER^+^/PR^+^/HER2^−^ TGCA BRCA patients^24^, resected >2 cm from the tumor margins, as well as their clinical information (Supplementary Dataset 6). Although only 1 tumor-adjacent normal sample per patient was available in the TCGA dataset, our multi-regional analyses demonstrate that breast cancer patients likely have a mixture of subtypes in the surrounding normal tissue. Hence, CIBERSORTx deconvolution was performed to estimate the proportion of each subtype within each tumor-adjacent normal sample using a signature matrix derived from the whole expression profile of our peri-tumoral samples (Supplementary Dataset 7, Methods). These samples were largely composed of Cluster 1-like and/or Cluster 2-like subtypes (Fig. 3d), while the basal-like Cluster 3 and skeletal muscle-enriched-Cluster 4 subtypes are rare.

As Clusters 1 and 2 are the most prevalent subtypes, we focused on these two subtypes and divided the TCGA breast cancer patients into Cluster 1- or Cluster 2-enriched groups based on their estimated proportions. Kaplan-Meier curves based on overall survival (OS) were plotted and patients with Cluster 1-enriched tumor-adjacent normal samples had significantly poorer OS by univariate and multivariate Cox regression analysis (adjusting for age, tumor size, lymph node involvement, and distant metastasis) compared to Cluster 2-enriched patients (Fig. 3e). Cluster 1 peri-tumoral features thus prognosticate poorer survival in breast cancer patients.

To ensure that this association was not specific only to the TCGA BRCA cohort, two additional microarray data sets (GSE31589 and GSE49175), consisting of 45 tumor-adjacent normal samples from ER^+^ breast cancer patients and 120 tumor-adjacent normal samples (tumor subtype not available) respectively were obtained^25,26^. These samples had been annotated using a previously reported Active or Inactive extratumoral subtype which was identified by unsupervised clustering of microarray data from 72 extratumoral breast tissues^25^. The Active subtype is significantly associated with poorer overall survival among ER^+^ breast cancer patients and characterized by EMT features, TGF-ß signaling, and inflammatory response. Although CIBERSORTx deconvolution estimated that over 90% of samples in both data sets had very high Cluster 1 proportions (data not shown), single-sample GSEA (ssGSEA) scores generated based on Cluster 1 and 2-enriched genes found earlier (Fig. 2c) showed that Active extratumoral subtype samples had significantly higher Cluster 1 ssGSEA score (*p* < 0.001) while the Inactive samples had significantly higher Cluster 2 ssGSEA score (*p* < 0.001) in both data sets (Fig. 3f, Supplementary Fig. 3g), supporting the survival analysis results from the TCGA BRCA cohort.

As such, Cluster 1-related features in the tumor-adjacent normal tissue are associated with worse clinical outcome while Cluster 2-features may have a protective effect in breast cancer patients.

### Differentially expressed genes and enriched pathways compared to non-tumor-bearing breasts

To understand why Cluster 1 subtype prognosticates poorer survival, we first examined differences in their transcriptomic profiles compared to non-tumor-bearing breast samples. Due to the stark contrasts in cell type composition between Clusters 2, 3, and 4 versus NTB samples (Fig. 2d), pathway enrichment analysis on differentially expressed genes (DEGs) identified similar enriched features as the analyses in Fig. 2c, highlighting differences in cell types present (Supplementary Fig. 4a–c, Supplementary Dataset 8).

**Fig. 4:**
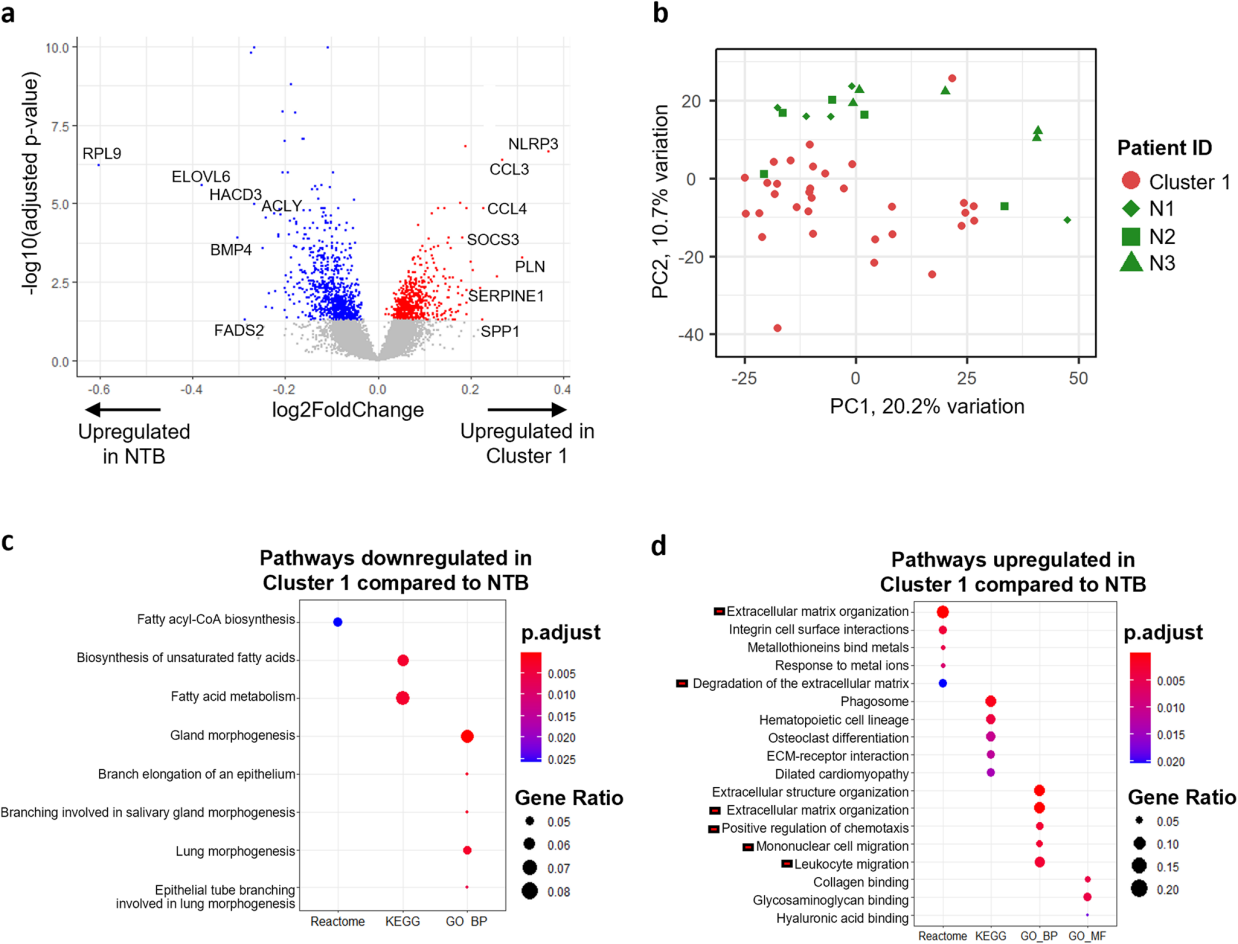
Differentially expressed genes and pathway activities between Cluster 1 and NTB samples. **a** Volcano plot showing the differentially expressed genes between Cluster 1 samples and NTB samples. Significantly upregulated genes in NTB are indicated in blue while significantly upregulated genes in Cluster 1 are indicated in red (adjusted *p* val < 0.05). Top downregulated genes based on fold-change and involved in fatty acid metabolism are labeled, while top upregulated genes with highest fold-change and involved in inflammation are labeled. **b** PCA plot of Cluster 1 and NTB samples. **c** Pathway enrichment results of top 100 genes downregulated in Cluster 1 peri-tumoral samples compared to NTB samples. **d** Pathway enrichment results of top 100 genes upregulated in Cluster 1 peri-tumoral samples compared to NTB samples.

On the other hand, although Cluster 1 had a similar cell type composition as NTB samples, 1451 DEGs were found (adj. *p* val < 0.05, no logFC threshold) (Fig. 4a, Supplementary Dataset 8). PCA performed on these samples alone also showed that Cluster 1 samples tend to cluster separately from NTB samples (Fig. 4b), despite overlapping in the PCA plot of all non-tumor samples in Fig. 2b. Pathway enrichment analysis on the top 100 downregulated genes in Cluster 1 peri-tumoral samples based on fold-change shows enrichment of fatty acid metabolism genes and gland morphogenesis compared to the NTB samples (Fig. 4c), whereas top 100 upregulated genes are involved in ECM degradation (e.g., *MMP9, MMP19, ADAMTS4*), as well chemotaxis regulation and leukocyte migration (e.g., *CCL3, CCL4, CCL8*) (Fig. 4d). This suggests increased ECM remodeling, immune cell recruitment, and inflammatory response in normal-appearing tissues surrounding the tumor. These 100 upregulated genes were also validated to be overexpressed in Cluster 1-enriched TCGA tumor-adjacent normal breast samples based on ssGSEA scores (Supplementary Fig. 4d).

As increased ECM-remodeling may contribute to cancer cell invasion while inflammatory cytokines may promote tumor growth and metastasis^27,28^, this provides a potential explanation for the poorer prognosis observed among breast cancer patients with Cluster 1-enriched NAT. Furthermore, this highlights that peri-tumoral tissues, though histologically normal, undergo gene expression changes influenced by the tumor which may further promote cancer progression.

### Cancer-associated fibroblast subtypes in peri-tumoral clusters

To better understand why these peri-tumoral subtypes prognosticate survival, we sought to further examine these peri-tumoral niches in terms of their stromal and immune compositions. While the previous analyses highlight differences in the main cell type constituents, fibroblasts and immune cells are the major TME components that have been reported by numerous studies to play critical roles in tumorigenesis and development^29^. However, studies on their profiles and functions in the peri-tumoral regions are lacking.

Cancer-associated fibroblasts (CAFs) have been recently characterized into two key subtypes by single-cell RNA-sequencing (scRNA-seq) studies^30–32^. Myofibroblast-like CAFs (myCAFs) express high levels of α-SMA (*ACTA2*) and upregulate genes involved in ECM organization and collagen formation, while inflammatory CAFs (iCAFs) upregulate the expression of cytokines and chemokines^30^. We thus sought to investigate whether these CAF subtypes were similarly found in peri-tumoral stroma and how they are spatially distributed in our patients.

**Fig. 5:**
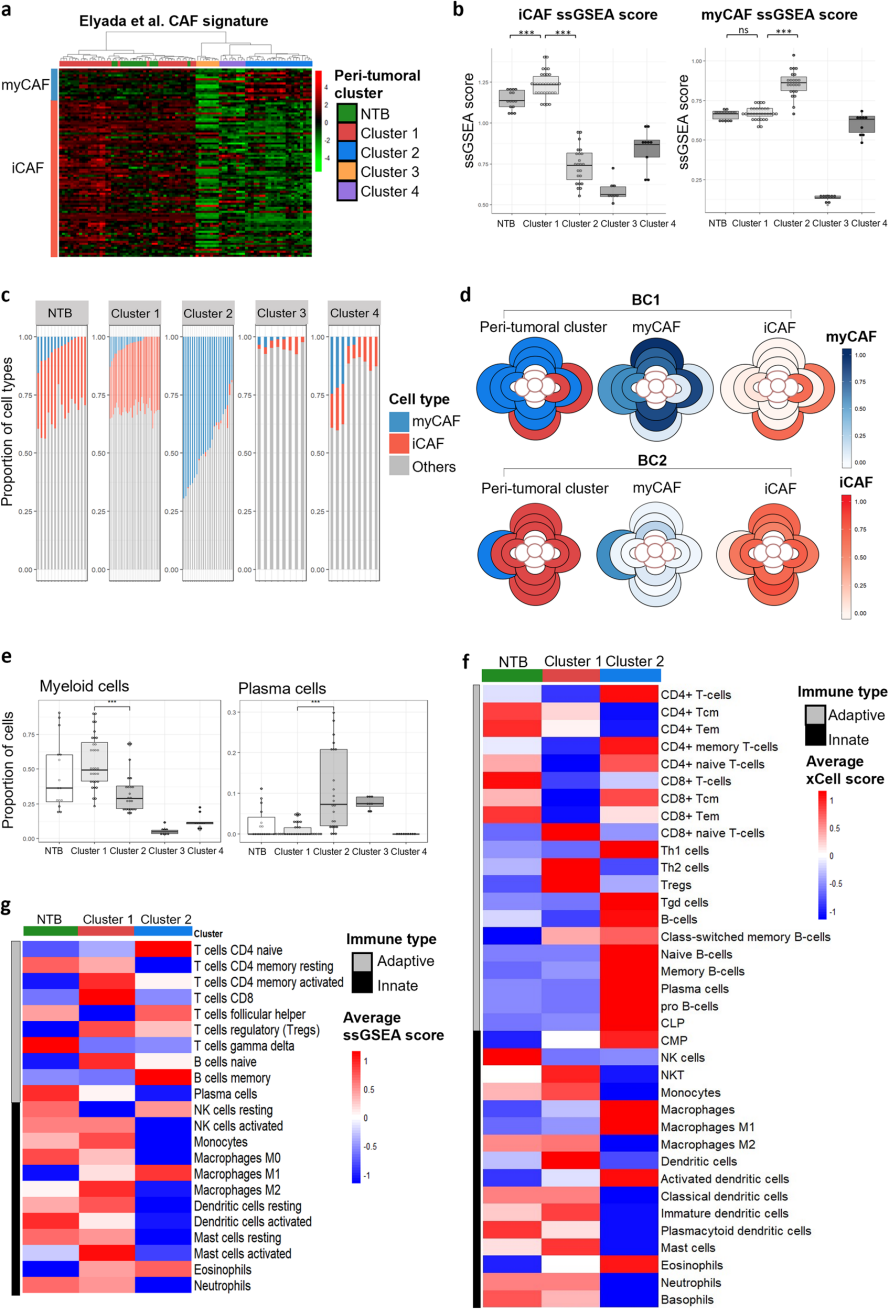
Cancer-associated fibroblast subtypes and immune cell compositions vary between peri-tumoral clusters. **a** Heatmap of myofibroblast-like CAFs (myCAFs) and inflammatory CAFs (iCAFs) signatures expressed by NTB and peri-tumoral samples (based on markers identified by scRNA-seq of human PDAC samples^30^). **b** ssGSEA scores for iCAF and myCAF marker genes for NTB and peri-tumoral clusters. Mann-Whitney *U* test p-values are shown. (***p ≤ 0.001). **c** Estimation of myCAF and iCAF proportions in NTB and peri-tumoral samples by CIBERSORTx deconvolution (using signature matrix built from scRNA-seq data of human PDAC samples^30^). **d** Representative spatial diagrams of 2 patients indicating cluster identity and estimated proportions of myCAFs and iCAFs in each region based on CIBERSORTx deconvolution. Scales represent relative estimated CAF subtype proportion within each sample. **e** Estimated proportions of myeloid cells, NK-T cells, and plasma cells in NTB and peri-tumoral samples by CIBERSORTx deconvolution (using custom matrix built from scRNAseq-data from Wu et al., 2020). Mann–Whitney *U* test *p* values are shown. (**p ≤ 0.01, ***p ≤ 0.001). **f** Heatmap of average xCell enrichment scores of 36 immune cell types for NTB, Cluster 1, and Cluster 2 samples. **g** Heatmap of average ssGSEA scores of 22 immune cell types for NTB, Cluster 1, and Cluster 2 samples.

Two methods were used to infer the CAF subtype abundance in the samples—an ssGSEA approach based on myCAF and iCAF marker genes identified from scRNA-seq on pancreatic ductal carcinomas^30^, as well as by CIBERSORTx deconvolution (Supplementary Dataset 9)^18^. Both approaches showed that NTB and Cluster 1 samples expressed significantly higher levels of iCAF marker genes and had higher estimated iCAF proportions, whereas Cluster 2 samples had higher myCAF ssGSEA scores and inferred proportions (Fig. 5a–c). Clusters 3 and 4 generally expressed very low levels of both myCAF and iCAF marker genes. These findings were similarly confirmed using another set of marker genes from scRNA-seq performed on triple-negative breast cancers (TNBCs) and by CIBERSORTx deconvolution (Supplementary Fig.s 5a–c, Supplementary Dataset 10).

To visualize the spatial distribution of CAF subtypes within the peri-tumoral regions of each patient, the spatial diagrams were re-plotted with each region colored based on their estimated myCAF and iCAF proportions (Supplementary Fig. 5d). Using two patients (BC1 and BC2) for illustration (Fig. 5d), we observed that their spatial distribution strongly reflects the cluster identity of each region. As such, no association with distance from the tumor was found, in contrast to previous studies that showed that myCAFs are found in close proximity to the invasive tumor interface while iCAFs are located more distally^31,33^. We further validated that Cluster 1-enriched TCGA tumor-adjacent normal samples similarly had significantly higher iCAF proportions while Cluster 2-enriched samples had higher myCAF proportions (Supplementary Fig. 5e).

Additionally, Kieffer et al. characterized CAFs into further subgroups based on clusters identified by scRNA-seq of FAP^Hi^CD29^med-hi^ fibroblasts from human breast cancers^34^. We applied these gene signatures to the peri-tumoral samples by generating ssGSEA scores for each subgroup and found that Cluster 1 samples were significantly associated with gene sets for all iCAF subgroups, namely IL-iCAFs, IFNγ-iCAFs, and detox-iCAFs, whereas Cluster 2 samples expressed high levels of ecm-myCAF and TGFβ-myCAF genes but not for wound healing-myCAFs (Supplementary Fig. 5f).

Lastly, as we observed that the NTB samples showed high levels of iCAF proportions and marker genes, we asked whether this was due to the samples coming from two *BRCA2* mutation carriers, which may have increased inflammatory signaling^35^. To this end, we obtained scRNA-seq data of normal breast tissue (*n* = 13) from Pal et al.^36^ and used the automated cell annotation package *SingleR* to classify the fibroblasts into myCAFs and iCAFs, using annotated scRNA-seq data from Wu et al.^31^ as the reference. Almost all fibroblasts in these reduction mammoplasty samples were classified as iCAFs (Supplementary Fig. 5g). This agrees with other studies that reported that iCAF-like fibroblasts were more abundant in healthy breast tissue by IHC and scRNA-seq whereas myCAF-like fibroblasts were sparse^31,37^, thus suggesting that our NTB samples are comparable in their fibroblast composition to other normal breast samples.

Taken together, our CAF-focused analyses suggest that the peri-tumoral clusters differ not only in their major cell type components, but in their fibroblast subtype composition as well. While fibroblasts located in adipose-enriched regions tend to be more iCAF-like, those in regions with higher smooth muscle and epithelial cell content are more myofibroblast-like. Given the heterogeneity in their properties and secretomes, these CAF subtypes likely impact tumor growth and development differently, further contributing to the difference in patient outcome observed between the peri-tumoral subtypes.

### Immune cell type composition in peri-tumoral clusters

The peri-tumoral regions not only consist of mesenchymal stromal cells, but also diverse immune cell types. As both Clusters 3 and 4 were previously inferred by ESTIMATE to have low immune cell infiltration (Supplementary Fig. 2f), further deconvolution into specific immune cell types is less reliable. As such, we focused on Clusters 1 and 2 which have more significant proportions of immune cells. Since ER^+^ breast cancers have been characterized by low immune cell infiltration^38^, we used three approaches to identify the most consistent and prominent patterns of immune cell composition among the samples.

CIBERSORTx deconvolution was used to estimate the proportions of eight broad immune cell types including myeloid cells, plasma cells, natural killer (NK) cells, and five T-cell subsets (Supplementary Dataset 10, Methods). While the proportions of T cells were generally similar between the clusters by this method (Supplementary Fig. 5h), significantly higher proportions of myeloid cells were identified in Cluster 1 (Fig. 5e). Next, we performed xCell cell type enrichment analysis^14^, which can infer the abundance of 36 immune cell types, including highly specific subsets and activation states (Fig. 5f and Supplementary Fig. 5i). We focused on 12 immune cell types based on the most significant p-value between clusters (Kruskal–wallis test) and greatest variance among all peri-tumoral samples (Supplementary Fig. 5j), and similarly found that Cluster 1 samples had higher myeloid cell abundance, specifically, neutrophils, monocytes, M2 macrophages, and inactivated dendritic cells (iDC). Lastly, we obtained a gene signature for 22 immune cell types from Xiao et al.^39^ to generate an ssGSEA score for each cell type (Fig. 5g and Supplementary Fig. 5k), which confirmed the enrichment of neutrophils, monocytes, M2 macrophages, and resting dendritic cells in Cluster 1 samples (Supplementary Fig. 5l).

On the contrary, Cluster 2 samples showed significantly higher proportions of plasma cells by both CIBERSORTx and xCell (Fig. 5e, f). Significant enrichment of other adaptive immune cells was also identified by xCell and ssGSEA, including CD4^+^ and CD8^+^ memory T cells, T helper 1 cells (Th1), gamma delta T cells (Tgd), T follicular helper cells (Tfh), and memory B cells (Fig. 5f, g, Supplementary Fig. 5j, l). Higher levels of activated dendritic cells (aDC) were also identified by xCell.

These three approaches to infer the abundance of different immune cell populations thus suggest a stronger innate immune response in Cluster 1-classified peri-tumoral regions compared to Cluster 2 samples, and a stronger adaptive immune response in Cluster 2-classfied regions compared both NTB and Cluster 1 samples. While further investigations are needed to understand why these immune cell types appear to co-localize with certain stromal cell types, these differences in immune cell composition may be another contributing factor for the prognostic value of the peri-tumoral subtypes in breast cancer patients.

## Discussion

**Fig. 6:**
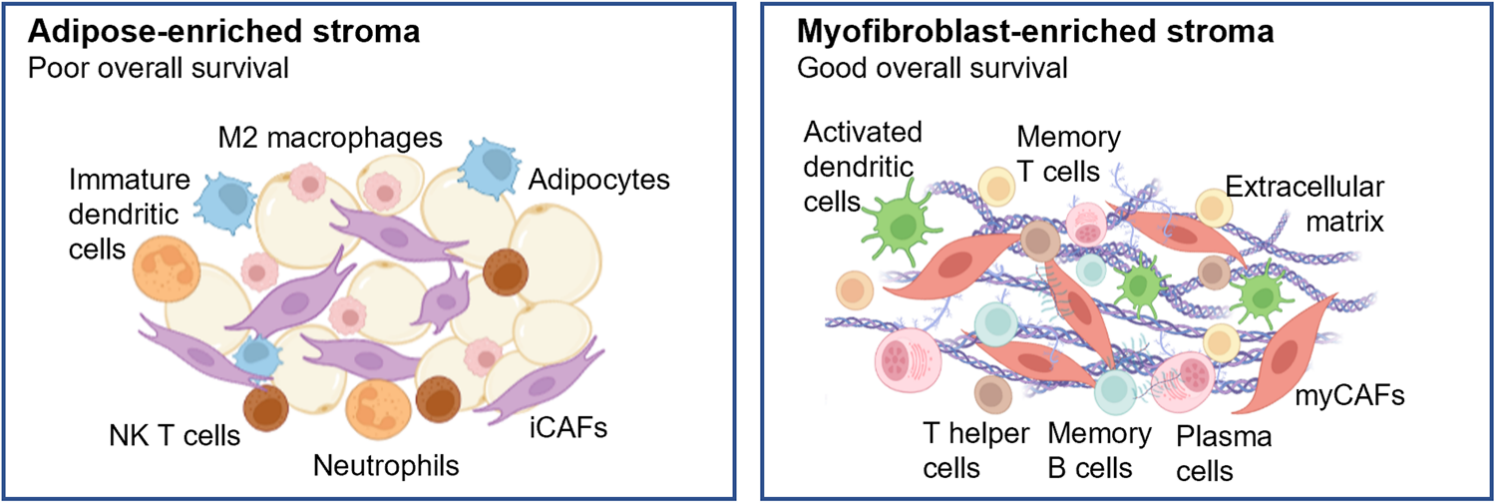
Graphical summary of Cluster 1 and 2 peri-tumoral subtypes.

In this study, we investigated the transcriptomic regional heterogeneity within breast tumors and peri-tumoral tissue in 8 breast cancer patients. We identified 4 peri-tumoral clusters, of which 2 clusters reflect the major breast stromal states (Fig. 6). The pro-inflammatory, adipose-enriched peri-tumoral subtype (i.e. Cluster 1) was significantly associated with poorer overall survival among breast cancer patients compared to the adaptive immune cell- and myofibroblast-enriched subtype (i.e. Cluster 2), indicating that the peri-tumoral stroma is not just an inert bystander of cancer progression, but may be actively involved in promoting or restraining tumor growth and development.

Interestingly, although we selected only ER^+^/PR^+^/HER2^−^-classified patients, molecular subtyping of individual tumor samples revealed 3 patients with basal-like tumor regions. Recent scRNA-seq studies on human breast cancers have also reported the presence of multiple subtype cells in tumors^31,37,40^. Although we did not observe any clinical outcome differences in these patients compared to those with only luminal tumor samples as yet, future longitudinal analysis could reveal the clinical consequences of multiple molecular subtypes within a tumor. Further study into the pathways regulating these molecular subtypes and how they develop during malignant transformation is needed to better incorporate them with conventional breast cancer classification systems.

Additionally, we identified 4 clusters from the peri-tumoral samples for which there appears to be a lack of spatial pattern in relation to the tumor. Instead, these clusters appear to be driven by underlying inter-patient and inter-regional differences in structural features and composition of the breast, including the proportion of fibroglandular and fat tissue in the breast, also referred to as mammographic breast density (MBD). Large inter-individual variations in MBD have indeed been well-established, ranging from almost entirely fatty (<25%) to extremely dense (>75%) breasts^41^. While MBD information was unavailable for patients in our cohort and in the TCGA dataset, Sun et al.^26^ previously reported that the Active extratumoral subtype, which is associated with poorer overall survival among ER^+^ breast cancer patients^25^ and also expressed higher levels of Cluster 1 genes (Supplementary Fig. 3g), was associated with significantly lower MBD than the Inactive subtype. These underlying host factors may thus drive each patient’s peri-tumoral subtype, independent of proximity to the tumor.

While MBD is also known to decrease with age^21^, we did not observe any correlation between age and peri-tumoral subtype within our 8 patients, likely since all patients are post-menopausal and above 50 years of age, although a positive trend between age and adipose-related gene expression was observed in a larger cohort of normal breast samples from healthy women (Supplementary Fig. 3d). Among our patients, we also did not observe any association between BMI and peri-tumoral subtype, nor among the normal breast samples (Supplementary Fig. 3e). Inconsistent results on the association between obesity and MBD appears have been reported thus far^42,43^, but future epidemiological studies with larger cohort sizes and different measures of body fatness may reveal new insights into how overall adiposity impacts breast adiposity.

While MBD is an established breast cancer risk factor^44^, reports on its role as a prognostic factor in breast cancer patients are less consistent and even contradictory^44–46^. In our analysis of TCGA breast cancer patients, those with tumor-adjacent normal samples enriched for Cluster 1 subtype had significantly poorer overall survival. We hypothesize that while the peri-tumoral subtype for each patient may be driven by host factors such as MBD, in the presence of a tumor, these pre-existing resident stromal cells are influenced differently and in turn lead to varying pro- or anti-tumorigenic effects.

As the adipose-enriched subtype prognosticates poorer overall survival, we hypothesize that this may be due to the presence of a pro-tumorigenic inflammatory environment, derived from the complex interplay between adipocytes, iCAFs, and innate immune cells present which co-localize in the same peri-tumoral region. Adipocytes, which are the main cell type constituent in this subtype, have been previously shown to secrete pro-inflammatory cytokines such as IL-1β, IL-6, and TNF-α^47^. While resident breast adipocytes may secrete a basal level of these cytokines to regulate normal breast physiology^47^, peri-tumoral adipocytes may be further induced by the tumor to upregulate the secretion of these pro-inflammatory factors, as seen in our Cluster 1 samples compared to NTB samples. Moreover, cytokines such as IL-1β and TNF-α have also been reported to be inducers of the inflammatory phenotype in CAFs^48^, which in turn upregulate the expression of cytokines and chemokines that may further promote tumor growth and proliferation^33,48^.

On the other hand, TCGA breast cancer patients with tumor-adjacent normal profiles expressing higher levels of Cluster 2-specific genes had improved overall survival. Although this gene set largely reflects the presence of luminal epithelial cells based on pathway enrichment analysis, our CAF-focused analyses demonstrate that a key stromal feature of these samples is the high myCAF proportion. Several in vivo mouse studies on pancreatic and colorectal cancer have indeed shown that depletion of αSMA^+^ myofibroblasts led to more aggressive tumors^49–52^, specifically through the production of type I collagen which may physically impede tumor-cell invasion^52,53^. Future studies are needed to further investigate the tumor-restraining functions of peri-tumoral myCAFs in breast cancer.

Interestingly, another key feature of Cluster 2 peri-tumoral samples is the enrichment of adaptive immune cell types. Besides myCAFs and iCAFs which have been identified by scRNA-seq of both human and mouse tumors, antigen-presenting CAFs (apCAFs) which express MHC class II genes have also been found in mouse pancreatic and mammary tumors^30,54^. Recently, Kerdidani et al. showed that apCAFs are frequent in human lung non-small cell carcinomas where they actively promote CD4-T-cell anti-tumor activity^55^. Further investigation into whether human breast tumors and peri-tumoral regions also contain apCAFs is needed and might provide an explanation for the increased adaptive immune response observed in these samples.

While we have provided an in-depth description of the transcriptomic heterogeneity in these eight patients, we acknowledge that there are limitations to this study. Firstly, due to extensive multi-regional sample collection, many of the peri-tumoral samples obtained were very small in size, leading to insufficient tissue amount and integrity for further histological validation or IHC staining of individual samples. Additionally, due to the limited availability of normal breast tissue specimens, NTB samples were obtained from 2 *BRCA2* mutation carriers, who may have altered transcriptomic profiles from wild-type healthy breast tissues. While we did not observe any striking differences between these samples and those from the wild-type-*BRCA2* reduction mammoplasty specimen, we acknowledge that these samples might have differences from normal healthy breast samples which we were unable to capture. We also acknowledge that our sample size of 8 cases is small and might not fully capture the full extent of peri-tumoral heterogeneity across all breast cancer patients. While we have included several publicly available gene expression data sets to validate our findings in this study, we hope that the peri-tumoral stromal gene signatures we have identified will be useful for future studies which may have access to larger cohorts of tumor-adjacent normal tissues and can be further studied for their correlation with other clinical variables (e.g., BMI, mammographic breast density).

Lastly, the use of bulk transcriptomics limited our ability to precisely compare the gene expression profiles between specific cell types, hence we instead focused on heterogeneity in cell type composition between the samples. Future work using scRNA-seq will better elucidate which factors secreted by the tumor are driving these changes in specific stromal and immune cells of the peri-tumoral environment. Spatial transcriptomics will also improve our understanding of how different stromal and immune cells are spatially organized and co-localize within the peri-tumoral compartments and how they interact with each other to impact tumor growth and development.

## Methods

### Sampling design

Surgically resected tissues were obtained from mastectomy specimens from 8 patients with primary breast cancer at the National Cancer Centre Singapore (NCCS). Patients had been identified prior to surgery and provided written informed consent for tissue harvest for the purpose of this study. This study has complied with all relevant ethical regulations including the Declaration of Helsinki and ethical approval was obtained from the National Cancer Centre Singapore Institutional Ethics Board (approval number: CIRB2015/2722). Six patients had invasive ductal carcinoma (IDC), 1 patient had mixed IDC and mucinous carcinoma, and 1 patient had invasive lobular carcinoma (ILC) (Supplementary Dataset 1). The age of patients ranged from 52 to 74 years (mean age = 62.3 ± 6.7 years). All patients had a unifocal breast carcinoma diagnosed pre-operatively with a macroscopic size of ≥2.0 cm. Free-hand dissection using sterile scalpel blades was performed to obtain five samples per tumor from different regions (center, superior, inferior, medial and lateral), as well as from non-pathological regions around the tumor to obtain peri-tumoral stromal samples (Fig. 1A). Three zones were identified around the tumor (Zone 1: 1–3 cm, Zone 2: 3–5 cm, Zone 3: 5–7 cm) based on macroscopic measurements from the tumor edge. Tissues within 1 cm macroscopic distance from the visible tumor were avoided to ensure only non-pathological breast tissue was collected. Within each zone, tissue was collected in four radial directions (superior, inferior, medial, or lateral) with an approximate size of 0.5 × 0.5 × 0.5 cm.

Standard IHC staining for estrogen receptor (ER), progesterone receptor (PR), and HER2 were performed on a single tumor sample per patient and all patients were classified as ER^+^, PR^+^, and HER2^−^ or equivocal based on standard IHC staining of a single tumor sample per patient (Supplementary Dataset 1). HER2 FISH (fluorescence in situ hybridization) testing was performed on patients who had equivocal HER2 status and confirmed that all tumors were HER2^-^. Standard H&E staining was also performed to determine the histological subtype, grade, and presence of fibrosis, stromal edema, and stromal tumor-infiltrating lymphocytes (TILs). As of January 2022, all patients are alive and well and only one patient had experienced local recurrence (BC4).

Surgically resected breast tissues of three NTB were obtained from a patient who had undergone breast reduction surgery for macromastia (N1) whereas patients N2 and N3 were germline *BRCA2*-mutation carriers who had undergone risk-reducing prophylactic surgery. For each patient, 5 non-directional samples were obtained.

### RNA extraction and DNA microarray

Total RNA was extracted using TRIzol reagent (Invitrogen) according to manufacturer’s instructions and homogenized using QIAGEN TissueLyser II. Extracted RNA was treated with DNase and purified on a Rneasy column (Qiagen). RNA quality and quantity were analyzed on a ND-1000 NanoDrop spectrophotometer and reverse transcribed using Superscript II (Invitrogen), before running on the human Clariom™ S Assay (ThermoFisher Scientific).

### Data analysis

#### Data pre-processing

The Clariom S Assay for human samples was used for transcriptome-wide gene-level expression profiling of tumor and stroma samples. Although we attempted to obtain 12 stromal samples per patient (4 radial directions × 3 distance zones), we were unable to do so for most patients, either due to the tumor location such that there was insufficient stromal tissue at the specified distance, or due to poor RNA quality. A total of 126 samples passed the quality control check (Tumor samples = 39, Peri-tumoral samples = 72, NTB samples = 15) for which the expression levels of 17507 genes were profiled. The R package Affy and rma function (Robust Multiarray Average) was used for quantile normalization and background correction.

#### PCA and clustering methods

PCA was performed on the expression values of all genes for tumor and stromal samples respectively using the R package PCAtools with the removal of the lowest 10% of variables based on variance. To validate the PCA clusters identified, two independent clustering techniques, K-means clustering and non-negative matrix factorization (NMF), were applied. The k-means function in R was used to compute k-means and the within-cluster sum of square (wss) for each k up to *k* = 20. The wss curve was plotted and the optimal number of clusters was selected by the elbow method. The NMF R package was used to confirm clustering patterns by plotting consensus matrices and obtaining the cophenetic coefficients for *k* = 2 to *k* = 6. Peri-tumoral samples were assigned to the 4 clusters using the k-means function in the cluster package.

#### Dendrograms and heterogeneity scores

Matrices of pairwise Manhattan distances were computed using the dist function in R between all tumor samples or all peri-tumoral samples for each patient respectively using the expression data for all genes. The ape package was used to plot the unrooted dendrograms based on the Manhattan distance matrices and edge lengths/1000 are labeled. A summary heterogeneity score is computed by taking the average of the pairwise Manhattan distances for all tumor or all peri-tumoral samples for each patient.

#### Molecular subtype classification

The genefu R package was use to classify the tumor samples into the molecular subtypes using the molecular.subtyping function and setting the sbt.model to “PAM50” or “AIMS”. As the PAM50 assignment may be affected by unequal distribution of ER^+^ and ER^−^ tumors in the cohort^56^, PAM50 was repeated on a larger dataset which combined both our tumor samples, which are likely to be mostly ER^+^, and another 66 ER^−^ microarray samples from TCGA BRCA patients. Tumor samples were then assigned to the molecular subtype called by at least two out of these three approaches.

#### Biological processes scoring

Stromal and immune scores were defined as the infiltrating stromal and immune cell scores as inferred by the ESTIMATE method^16^ using the estimate R package. Scores for all other biological processes were calculated using a Z scoring approach^15^ based on gene sets representing each biological process. Genes annotated by the GO term GO:0008283 were used for the proliferation score while genes annotated by the GO term GO:0001525 were used for the angiogenesis score. The HALLMARK_APOPTOSIS gene set was used for the apoptosis score while a hypoxia signature from Buffa et al.^57^ was used for the hypoxia score. For each gene in the ith tumor or stromal region, its *Z* score is calculated as Z_I_ = (*e*_i_–*µ*)/*σ*, where *e*_i_ is its expression, *µ* and *σ* are mean and standard deviation respectively from the 15 NTB samples. For each sample, the final score for each biological process is calculated by taking the average *Z* score for all genes in its associated gene set. For EMT, a list of 170 epithelial genes and 48 mesenchymal genes was obtained from Tan et al.^58^, and EMT score = average *Z* score of mesenchymal genes—average *Z* score of epithelial genes. All scores were normalized to between 0 and 1 before plotting.

#### Differential gene expression, pathway enrichment analysis, and GSEA

The limma R package was used to identify DEGs (Log2FC > 0.5 or 1, adjusted p-val<0.05). Pathway enrichment analysis was performed using the R package ReactomePA^59^ for analysis using the Reactome database while the clusterProfiler package^60^ was used for analysis based on the Kyoto Encyclopedia of Genes and Genomes (KEGG) and Gene Ontology (GO) databases. The p-value cut-off was set to 0.05 and q-value cut-off was set to 0.2 in all analyses and top 5 enriched pathways were plotted. GSEA was performed using the GSEA desktop application (v4.1.0). Single-sample gene set enrichment analysis (ssGSEA) was performed using the GSVA R package with method set to “ssgsea”. All heatmaps were plotted using the pheatmap package.

#### Cell type enrichment and deconvolution analysis

The xCell webtool was used to perform cell type enrichment analysis to obtain enrichment scores for 64 cell types which represent their abundance in the bulk gene expression sample (Supplementary Dataset 2). A full list of abbreviations for the 64 cell types can be found here^61^. The CIBERSORTx tool was used to estimate the proportion of immune cell types in tumor samples using the LM22 signature matrix and run in absolute mode. For the deconvolution of peri-tumoral samples, we obtained a signature matrix for 34 cell types^18^, based on an original reference panel of 250 RNA-seq samples. The matrix was reduced to 28 cell types by removing certain non-relevant cell types (eg. neurons, astrocytes) and used to run the deconvolution step in relative mode (Supplementary Dataset 5). Another signature matrix was also adapted from Li et al.^18^ for deconvolution of CAF subtypes, which was built from PDAC scRNA-seq data from Elyada et al.^30^ (Supplementary Dataset 9). Annotated TNBC scRNA-seq expression data from Wu et al.^31^ was used to create a second custom signature gene matrix for the deconvolution of CAF subtypes and immune cell types (Supplementary Dataset 10). The top 250 genes upregulated in myCAFs and iCAFs as reported by Wu et al.^31^ were used as marker genes for each CAF subtype for heatmap plotting. The whole expression profiles of our peri-tumoral samples were used to create a custom signature gene matrix for deconvolution of TCGA tumor-adjacent normal samples (Supplementary Dataset 7). For all analyses, the number of permutations for significance analysis was set to 100.

#### Validation data sets

Bulk RNAseq expression data from normal adjacent to tumor (NAT) tissues from ER^+^/PR^+^/HER2^−^ TCGA breast cancer patients and their clinical information (*n* = 50) as well as RNA-seq data of normal breast samples from 145 healthy women without breast disease from Kang et al.^23^ were obtained from UCSC Xena^24^ (https://xenabrowser.net/datapages/). Microarray data of extratumoral normal tissue and annotation for active/inactive subtype was obtained from GSE31589 and GSE49175 using the Geoquery R package. GSE31589 consists of 72 tumor-adjacent normal samples that were previously found to express either an extratumoral Active or Inactive subtype^25^, of which 45 samples from ER^+^ patients were used in the analysis. GSE49175 consists of 120 tumor-adjacent normal samples from the Polish Women’s Breast Cancer Study (ER, PR, HER2 status not available) which were similarly classified according to this Active/Inactive subtype^26^. scRNA-seq expression data of normal breast tissue was obtained from Pal et al. (GSE161529)^36^. The SingleR package was used to perform automated cell type annotation using annotated scRNA-seq data from Wu et al.^31^ as reference.

#### Survival analysis

The survival and survminer R packages were used to perform univariate and multivariate Cox regression analysis in the TCGA BRCA cohort. The patients were divided into Cluster 1-enriched and Cluster 2-enriched groups based on whether their Cluster 1 or Cluster 2 CIBERSORTx inferred proportions were above or below the median of the cohort. Multivariate Cox regression model includes age and TNM staging, in addition to Cluster 1 or 2 proportions. Updated overall survival data of TCGA patients generated by Liu et al.^62^ was used.

#### Statistical analysis and software used

All graphs and statistical analyses were performed using R version 3.6.1 or GraphPad Prism version 8.4.3. Statistical significance was assessed using Mann–Whitney test and Kruskal–Wallis test as applicable. Figures 1a, 6 and Graphical abstract were created with BioRender (license number CK25OOQB77).

### Reporting summary

Further information on research design is available in the Nature Research Reporting Summary linked to this article.

### Supplementary information

Supplementary material

Reporting summary

Supplementary Dataset 1

Supplementary Dataset 2

Supplementary Dataset 3

Supplementary Dataset 4

Supplementary Dataset 5

Supplementary Dataset 6

Supplementary Dataset 7

Supplementary Dataset 8

Supplementary Dataset 9

Supplementary Dataset 10

## Data Availability

Raw CEL files and processed expression data have been deposited as GEO accession number GSE211729. Whole-slide H&E images for samples that had sufficient tissue (39 tumors and 50 peri-tumoral samples) are available from the authors upon request.

## Code availability

All code used for analysis and plotting is available from the authors upon request.
