# Supplementary material for: Adipose-enriched peri-tumoral stroma, in contrast to myofibroblast-enriched stroma, prognosticates poorer survival in breast cancers
Source: NPJ Breast Cancer. 2023 Oct 20;9:84. doi: 10.1038/s41523-023-00590-7 (PMC10589339; doi:10.1038/s41523-023-00590-7)
Supplement: Supplementary file 1 — Supplementary material [file 41523_2023_590_MOESM1_ESM.pdf]

Supplementary Material

Lau *et al.* - Adipose-enriched peri-tumoral stroma, in contrast to myofibroblast-enriched stroma, prognosticates poorer survival in breast cancers

Supplementary Dataset 1 – Clinical and pathological characteristics of the cohort.

Supplementary Dataset 2 – xCell enrichment scores of all samples.

Supplementary Dataset 3 – Biological processes scores and CAF subtype proportions for all samples.

Supplementary Dataset 4 – Significantly upregulated genes in each cluster compared to all other peri-tumoral samples.

Supplementary Dataset 5 – CIBERSORTx deconvolution proportions of 28 cell types and signature gene matrix used.

Supplementary Dataset 6 – Clinical data of TCGA patients analyzed.

Supplementary Dataset 7 – CIBERSORTx signature gene matrix used for deconvolution of TCGA tumor-adjacent normal samples into four peri-tumoral subtypes.

Supplementary Dataset 8 Differentially expressed genes between each cluster compared to NTB samples.

Supplementary Dataset 9 – CAF subtype marker genes from Elyada *et al.* (2019) and CIBERSORTx signature gene matrix derived from the corresponding scRNA-seq data.

Supplementary Dataset 10 – CAF subtype marker genes from Wu *et al.* (2020) and CIBERSORTx signature gene matrix derived from the corresponding scRNA-seq data.

Supplementary Figure 1a-f – Lau et al.

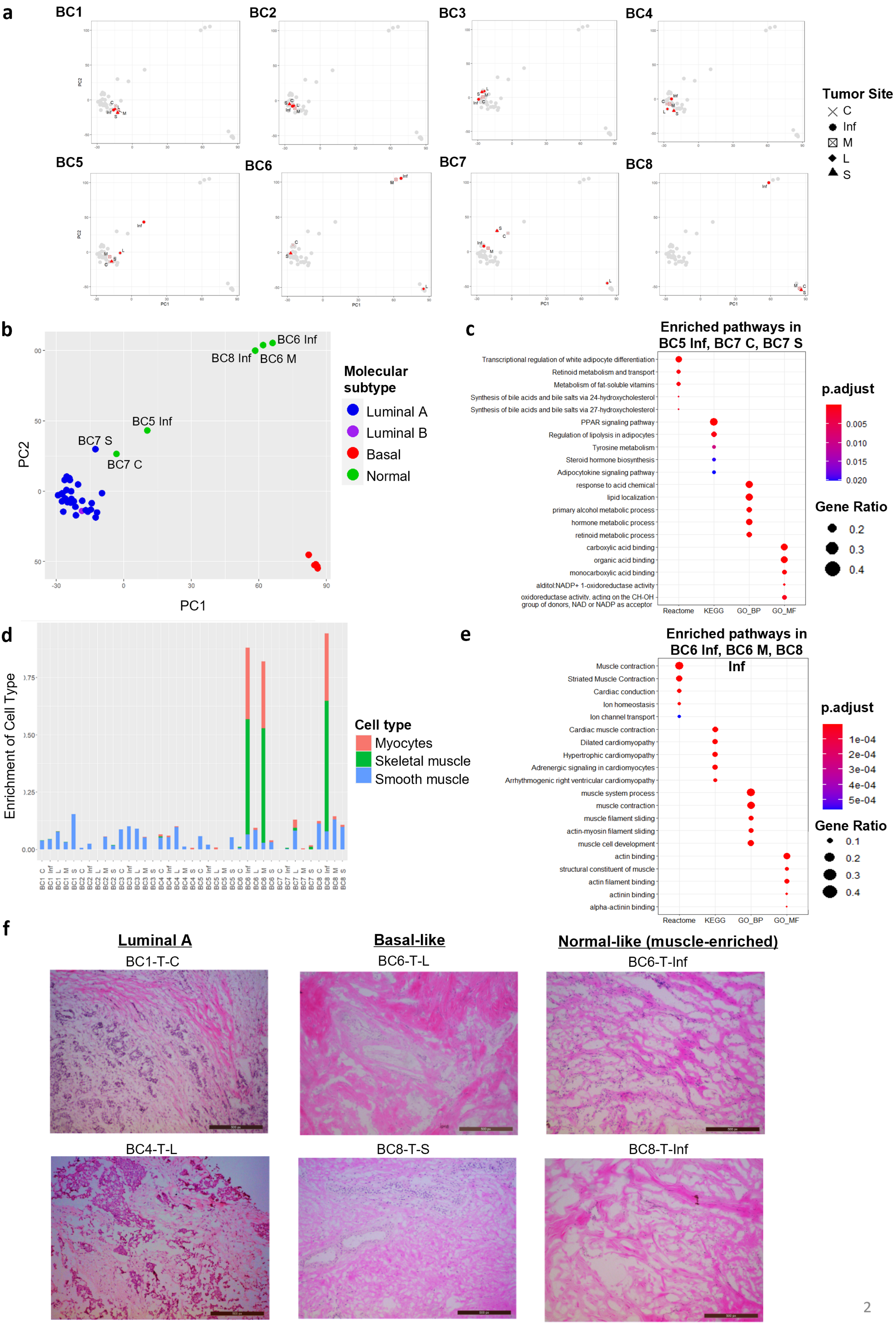

Supplementary Figure 1g-i – Lau et al.

g

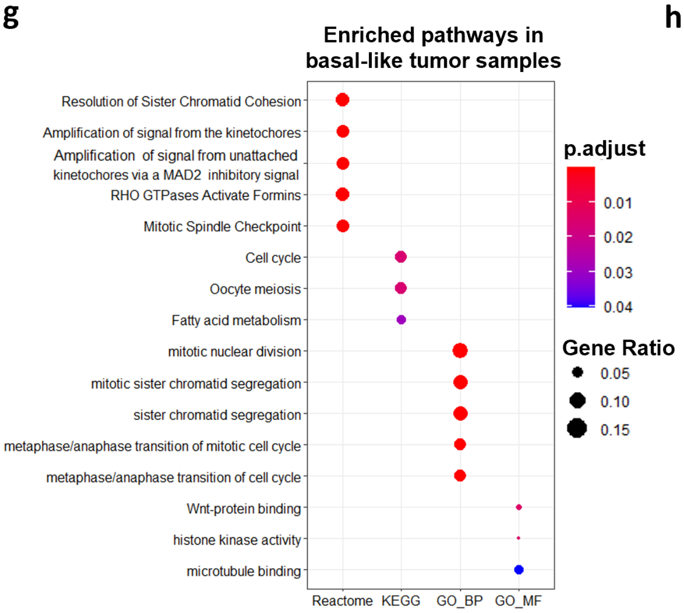

h

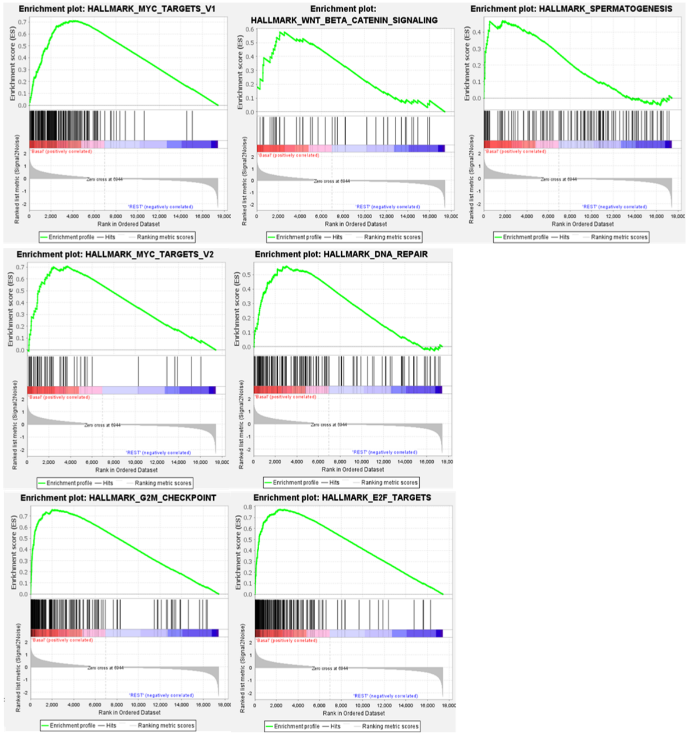

i

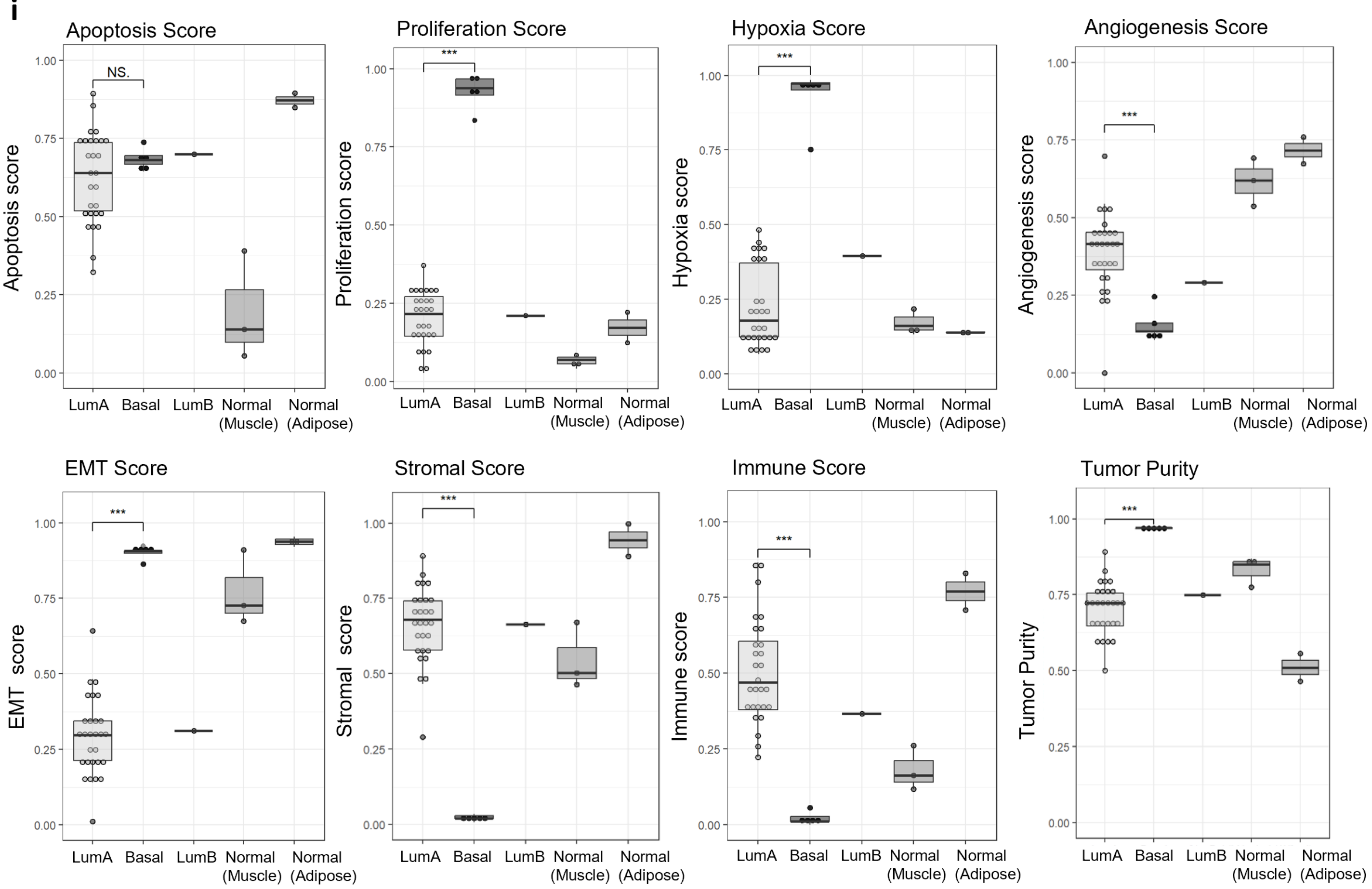

Supplementary Figure 1j – Lau et al.

j

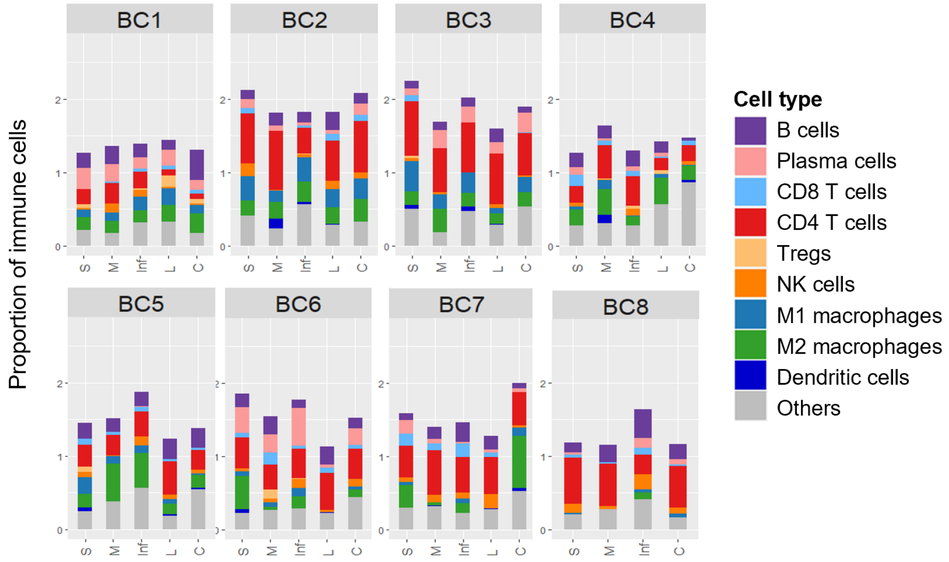

Supplementary Figure 1. Tumor samples from ER+/PR+/HER2- patients show inter-tumor and intra-tumor heterogeneity. (a) PCA plots of tumor samples as in Fig. 1B with each individual patient's samples indicated in red and all other samples in gray. **(b)** PCA plots of tumor samples as in Fig. 1B with each sample highlighted by its molecular subtype. **(c)** Pathway enrichment results for genes significantly upregulated in tumor samples BC5 Inf, BC7 S and BC7 C (logFC>1, adjusted p-value<0.05, top 5 results from each database shown). **(d)** xCell cell type enrichment scores of muscle-related cell types in tumor samples. **(e)** Pathway enrichment results for genes significantly upregulated in tumor samples BC6 Inf, BC6 M, BC8 Inf (logFC>1, adjusted p-value<0.05). **(f)** Representative H&E images of tumor samples for each molecular subtype (10X magnification). H&E images of adipose-enriched normal-like tumor samples could not be obtained due to low tissue integrity and quality of cryo-preserved samples. **(g)** Pathway enrichment results for top 100 genes significantly upregulated in basal-like tumor samples. **(h)** GSEA results for basal-like tumor samples compared to all other tumor samples at FDR<25% using 50 Hallmark gene sets. **(i)** Box plots showing scores for key biological processes by molecular subtype of tumor samples. Center line represents the median, box limits show the upper and lower quartiles and whiskers show 1.5x interquartile ranges. Mann-Whitney U test p-values are shown (*** $p \leq 0.001$). **(j)** Bar plots showing the proportions of immune cell types estimated by CIBERSORTx in tumor samples.

Supplementary Figure 2a-c – Lau et al.

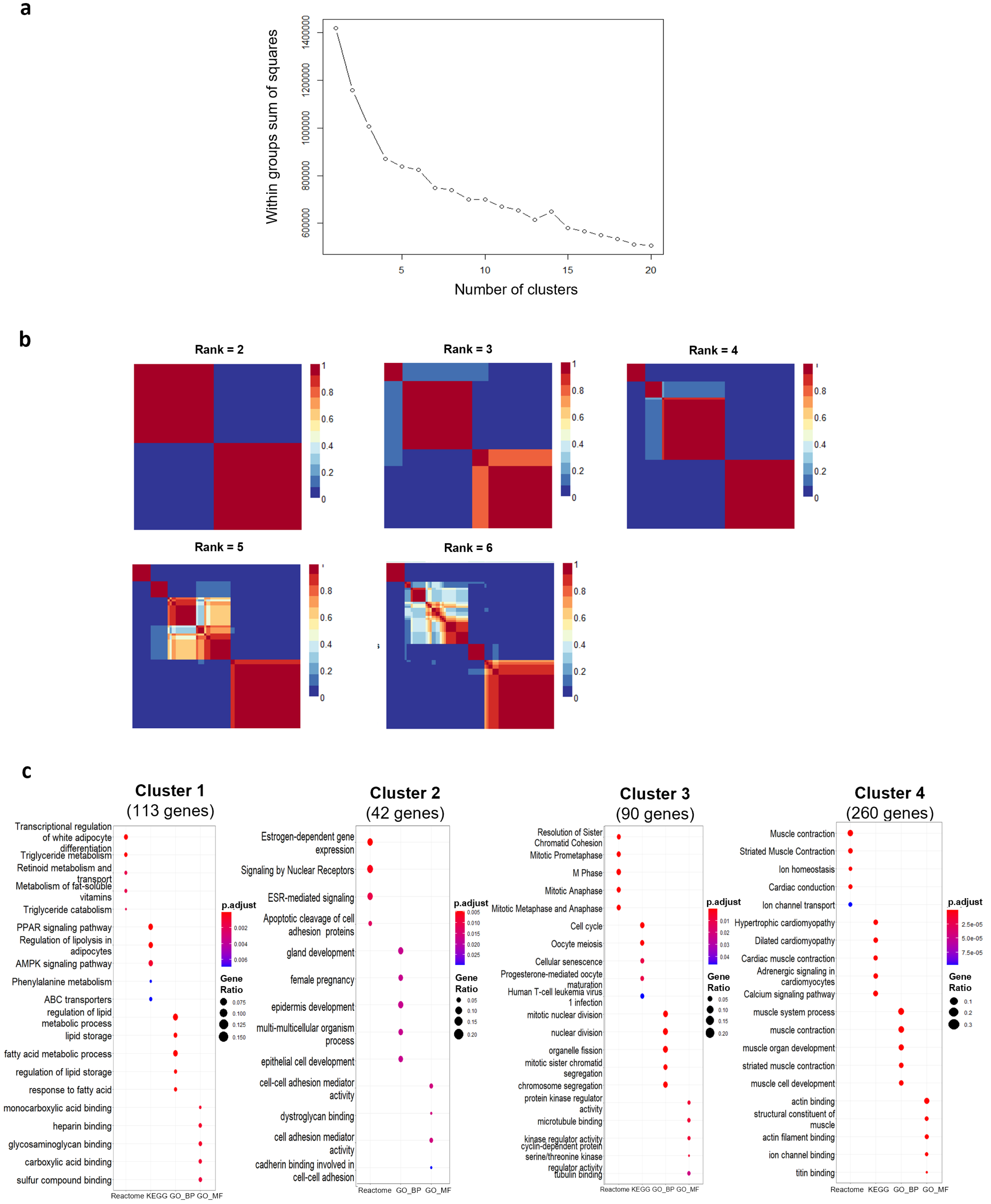

Supplementary Figure 2d-e – Lau et al.

d

**Cluster 1**	**Cluster 2**	**Cluster 3**	**Cluster 4**
BC1-St-M3	BC1-St-L2	BC6-St-S1	BC6-St-Inf2

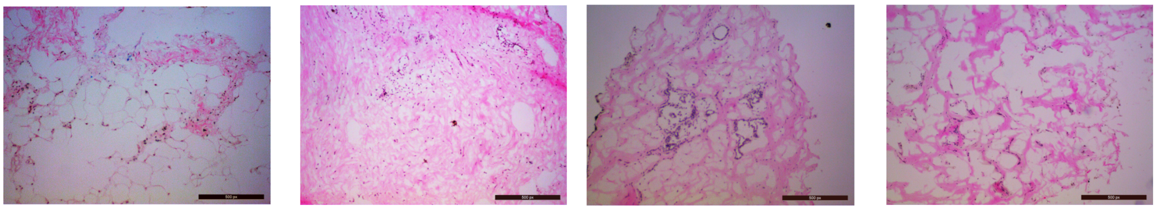

BC2-St-L1	BC1-St-M2	BC6-St-L3	BC4-St-L3

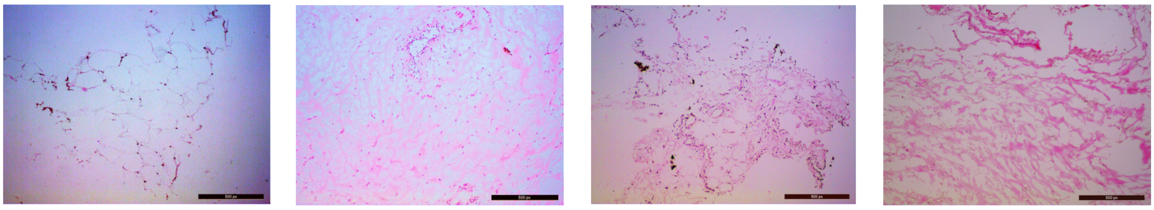

e

Cluster 1 vs Rest (4 gene sets upregulated)

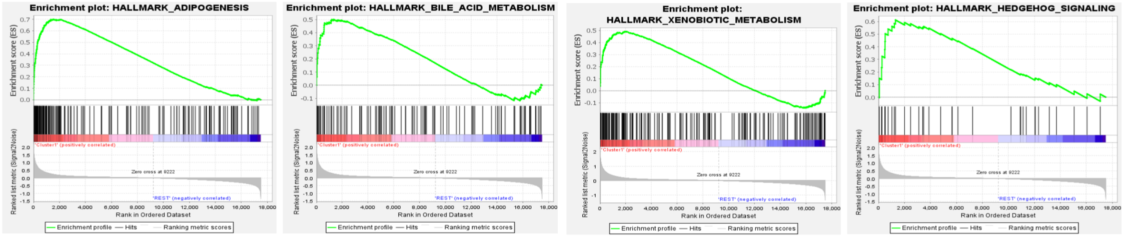

Cluster 2 vs Rest (1 gene set upregulated)

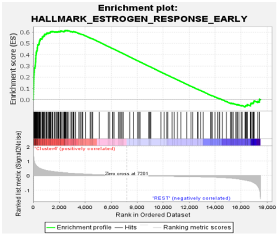

Cluster 3 vs Rest (12 gene sets upregulated)

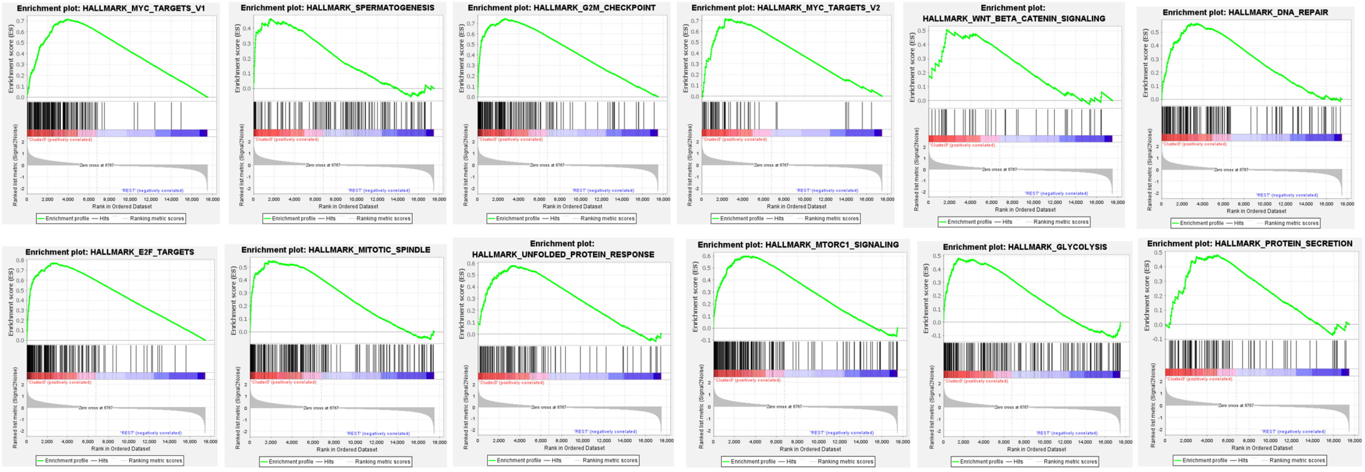

Cluster 4 vs Rest (3 gene sets upregulated)

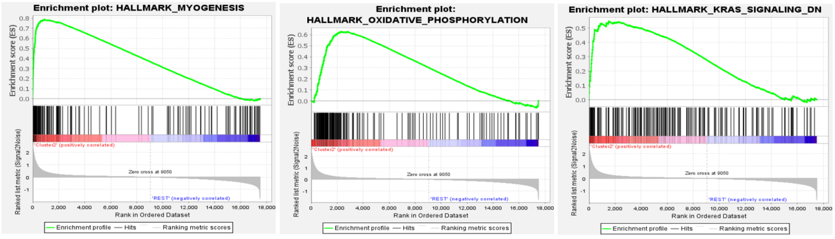

Supplementary Figure 2f – Lau et al.

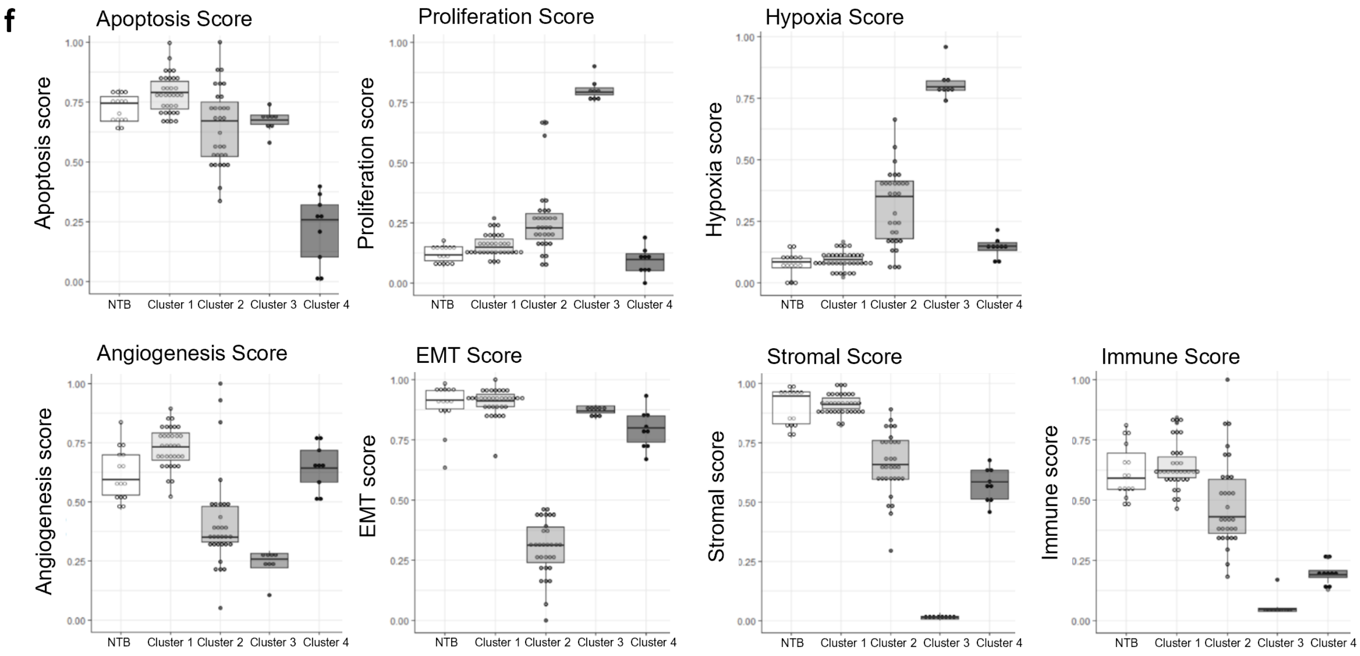

Supplementary Figure 2. Four clusters are identified from morphologically normal peri-tumoral samples in breast cancer patients. (a) Within-group sum of squares plot from K-means clustering of peri-tumoral samples shows that the optimal number of clusters is four. **(b)** Consensus matrices from NMF clustering of stromal samples shows that the most stable number of clusters is four (NMF cophenetic coefficient = 0.9998). **(c)** Pathway enrichment results of upregulated genes from each cluster compared to all other stromal samples (top 5 results by gene ratio from each database shown). **(d)** Representative H&E images of peri-tumoral samples from each cluster (10X magnification). **(e)** GSEA results of stromal samples from each cluster compared to all other stromal samples at FDR<25% using 50 Hallmark gene sets. **(f)** Box plots showing scores for key biological processes for NTB and stromal clusters. Center line represents the median, box limits show the upper and lower quartiles and whiskers show 1.5x interquartile ranges.

Supplementary Figure 3 – Lau et al.

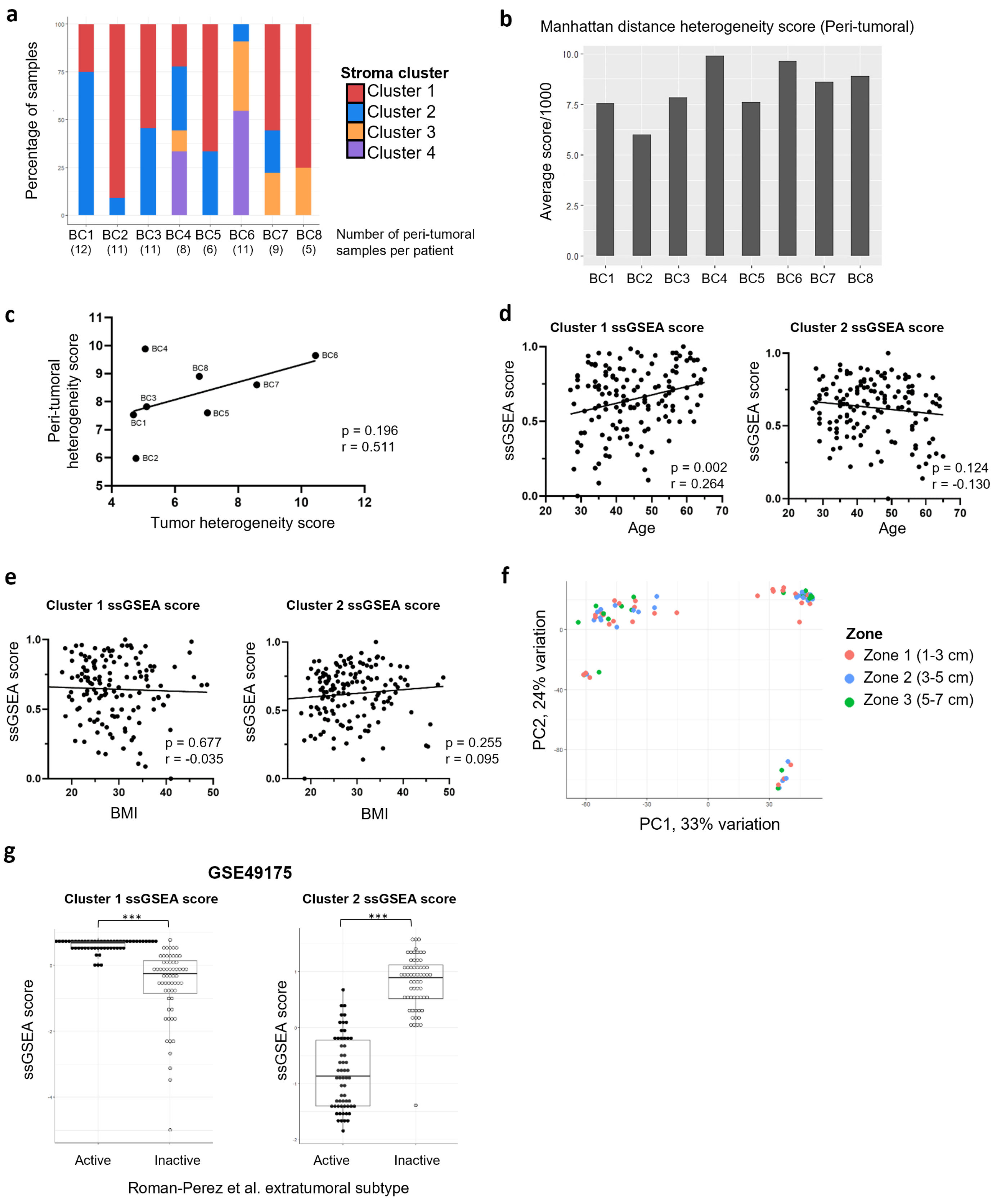

Supplementary Figure 3. Spatial distribution and prognostic value of peri-tumoral clusters in breast cancer patients. (a) Bar plots showing the percentage of peri-tumoral samples assigned to each cluster by K-means clustering for each patient. (continued on next page)

(b) Bar plot showing the heterogeneity score for each patient's peri-tumoral samples, calculated by taking the average pairwise Manhattan distances for all peri-tumoral samples for each patient. **(c)** Correlation between peri-tumoral heterogeneity score and tumor heterogeneity score for each patient. Pearson correlation r and p-value are indicated. **(d)** Correlation between age and Cluster 1-ssGSEA or Cluster 2-ssGSEA scores in normal breast samples from healthy women without breast disease from Kang *et al.* (2020) (n=145). Pearson correlation r and p-value are indicated. **(e)** Correlation between BMI (kg/m^2) and Cluster 1-ssGSEA or Cluster 2-ssGSEA scores in normal breast samples from healthy women without breast disease from Kang *et al.* (2020) (n=145). Pearson correlation r and p-value are indicated. **(f)** PCA plot of peri-tumoral samples coloured by distance zone (Zone 1: 1-3 cm, Zone 2: 3-5 cm, Zone 3: 5-7 cm from tumor edge). **(g)** ssGSEA scores for Cluster 1 and Cluster 2 genes in tumor-adjacent normal samples from GSE49175 (n=120), grouped according to Roman-Perez *et al.* annotation for Active/Inactive extratumoral microenvironment subtype. Mann-Whitney U test p-values are shown (*** $p \leq 0.001$).

Supplementary Figure 4a-c – Lau et al.

a

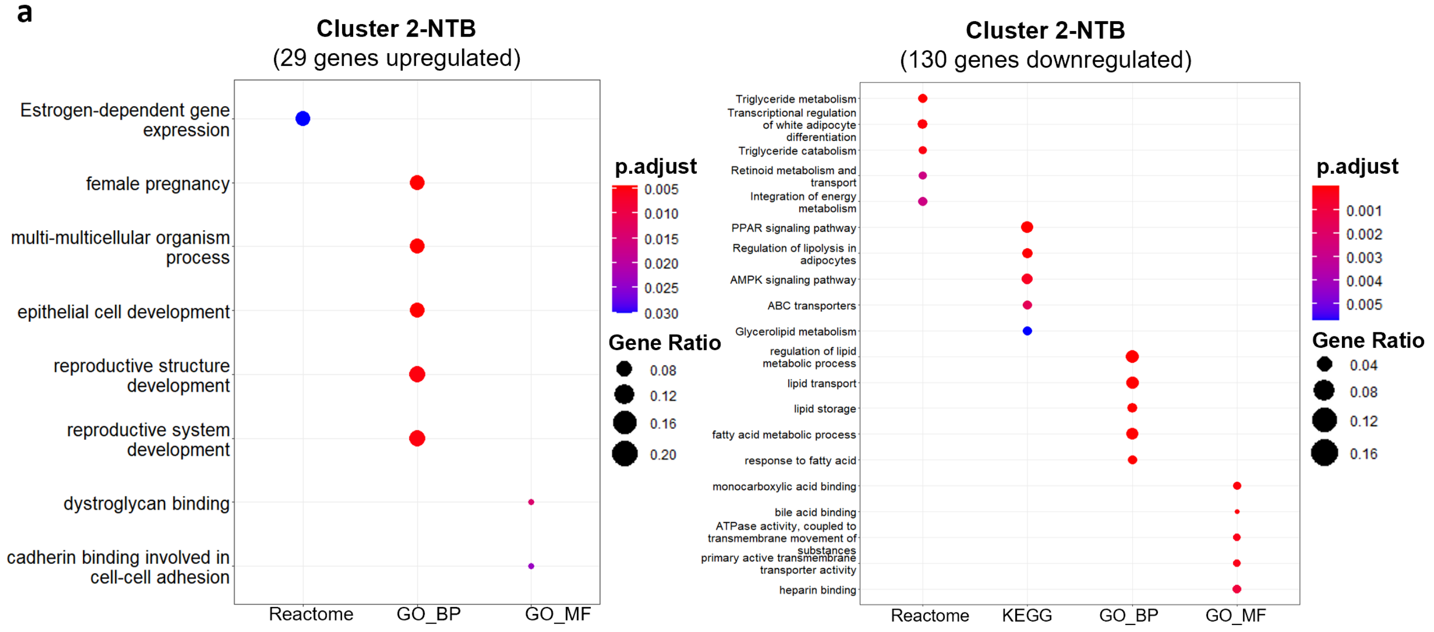

b

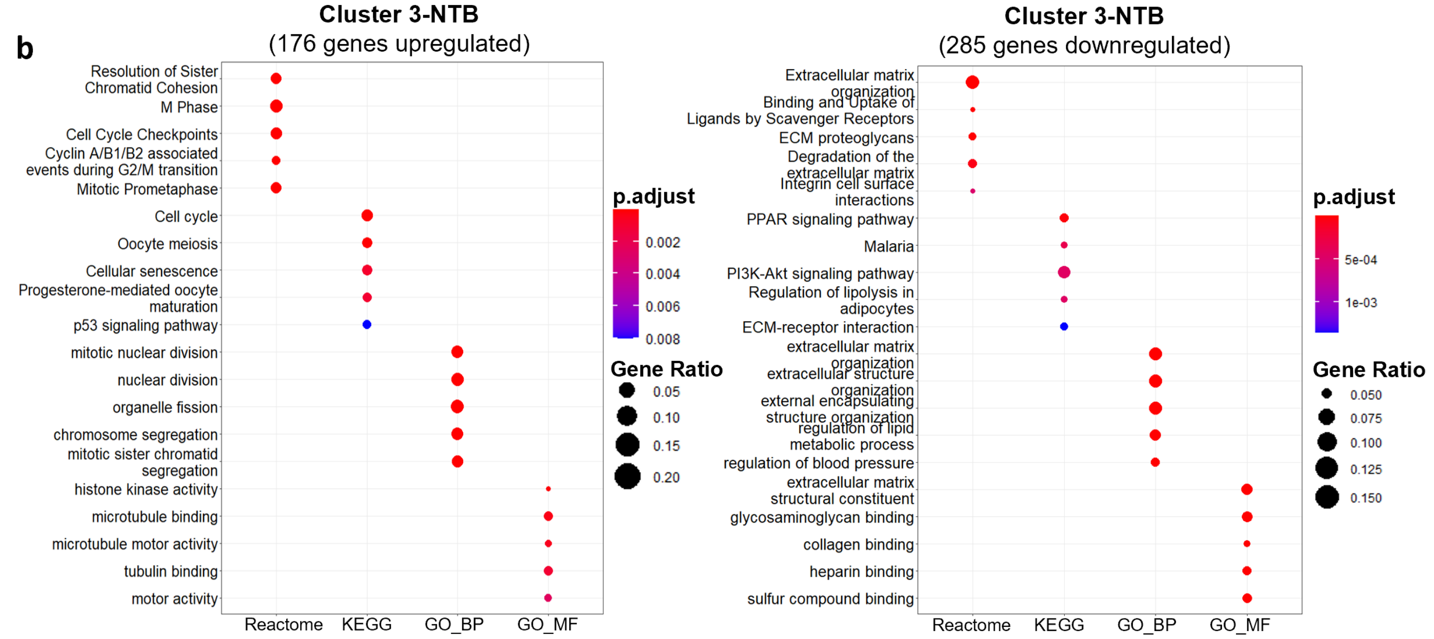

c

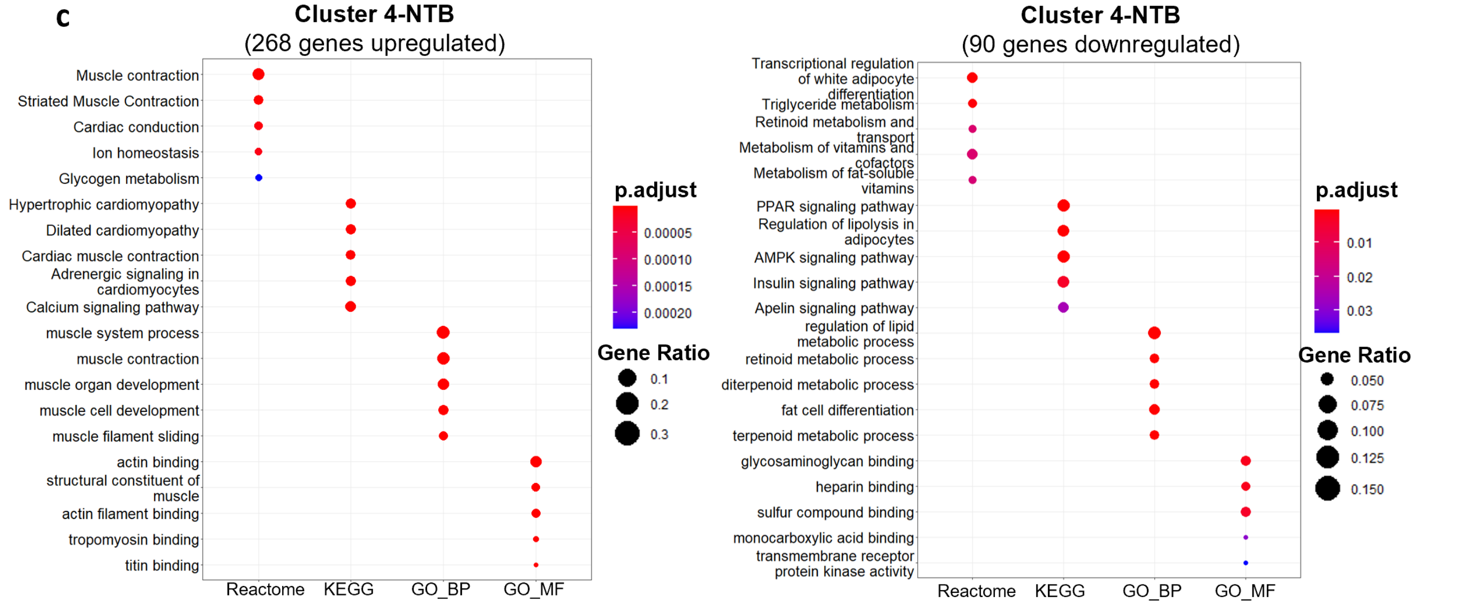

Supplementary Figure 4d – Lau et al.

d

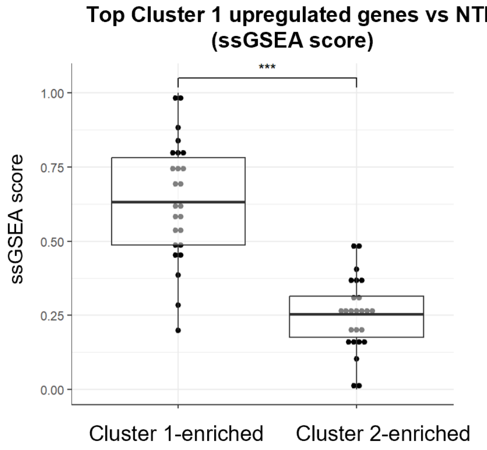

Supplementary Figure 4. Differentially expressed genes and pathway activities between Cluster 1 and NTB samples. (a) Pathway enrichment results of differentially expressed genes between Cluster 2 peri-tumoral samples and NTB samples (LogFC>0.5, adjusted p<0.05). **(b)** Pathway enrichment results of differentially expressed genes between Cluster 3 peri-tumoral samples and NTB samples (LogFC>0.5, adjusted p<0.05). **(c)** Pathway enrichment results of differentially expressed genes between Cluster 4 peri-tumoral samples and NTB samples (LogFC>0.5, adjusted p<0.05). **(d)** ssGSEA scores for top 100 genes upregulated in Cluster 1 compared to NTB samples in TCGA tumor-adjacent normal samples divided into Cluster 1-enriched and Cluster 2-enriched groups based on CIBERSORTx deconvolution proportions (n=50). Mann-Whitney U test p-values are shown (*** p ≤ 0.001).

Supplementary Figure 5a-c – Lau et al.

a

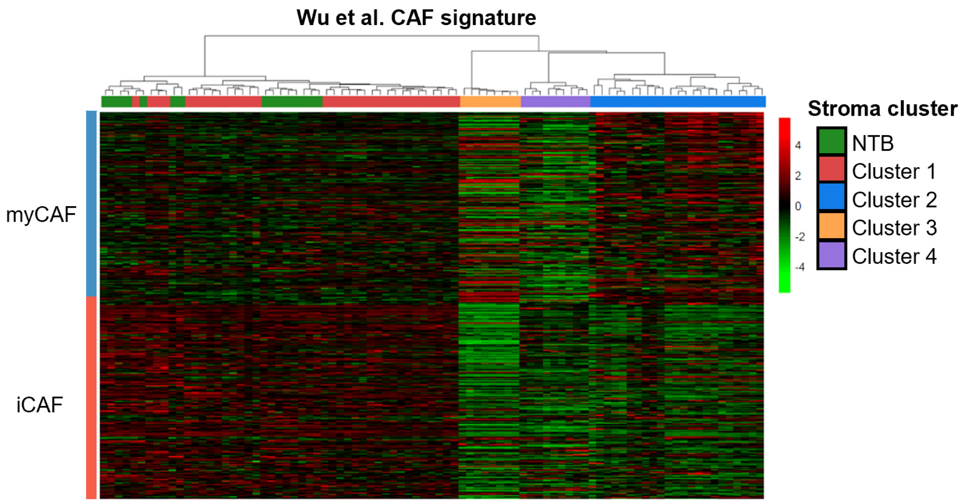

b

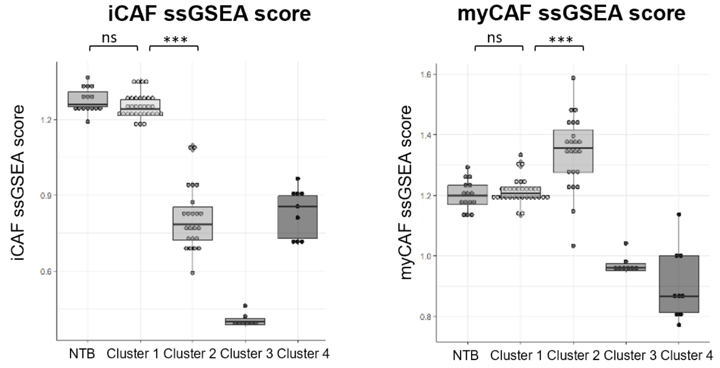

c

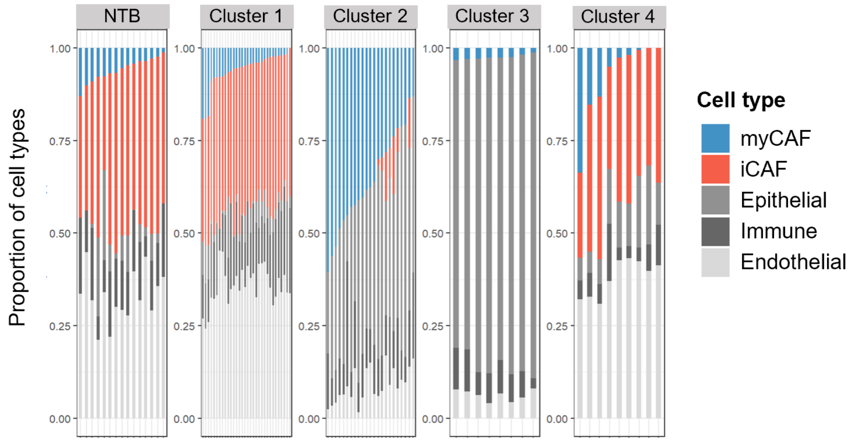

Supplementary Figure 5d-e – Lau et al.

d

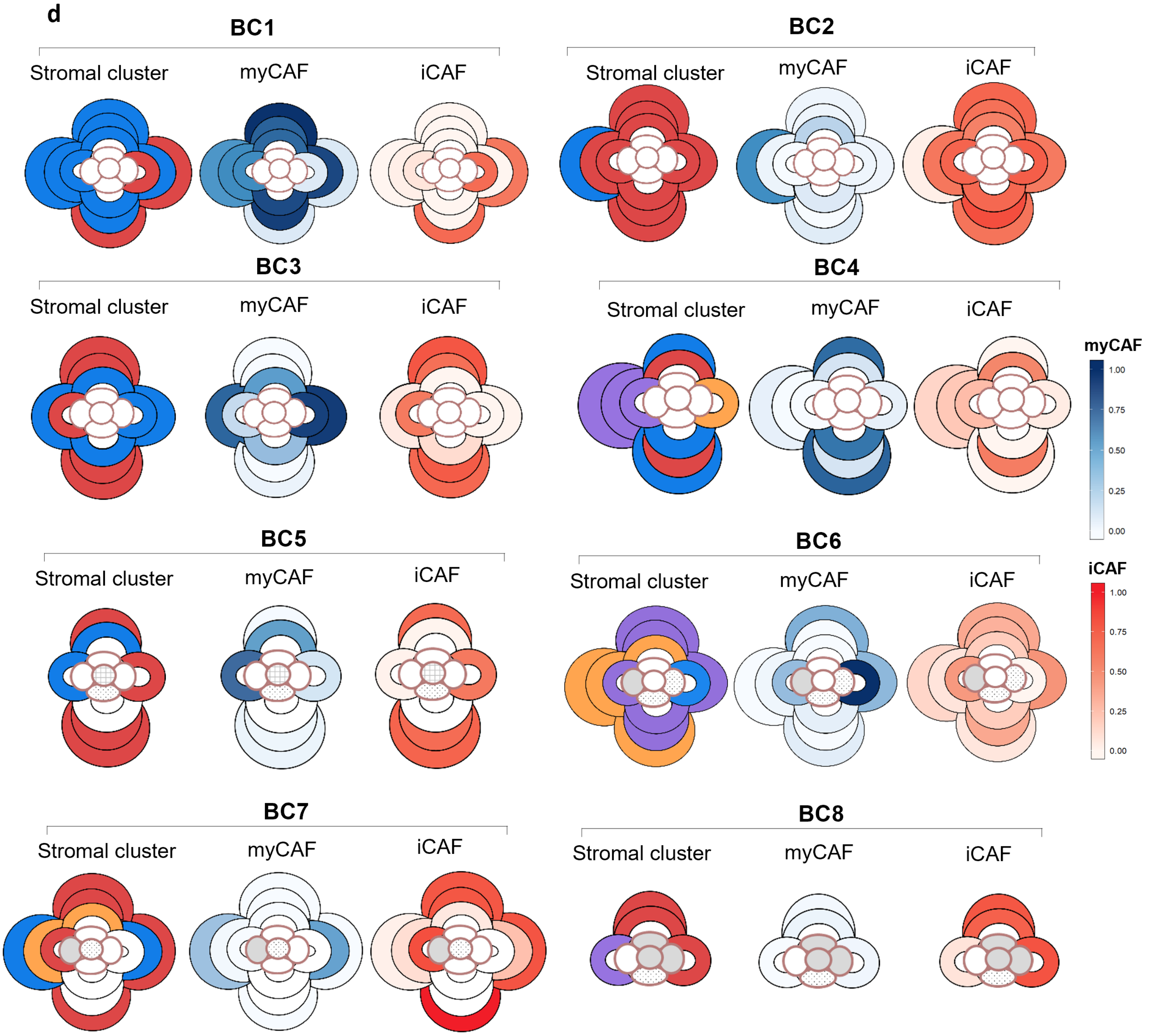

e

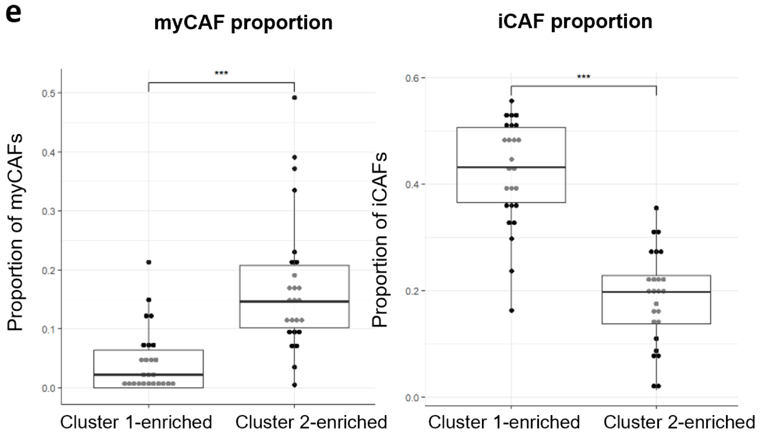

Supplementary Figure 5f-h – Lau et al.

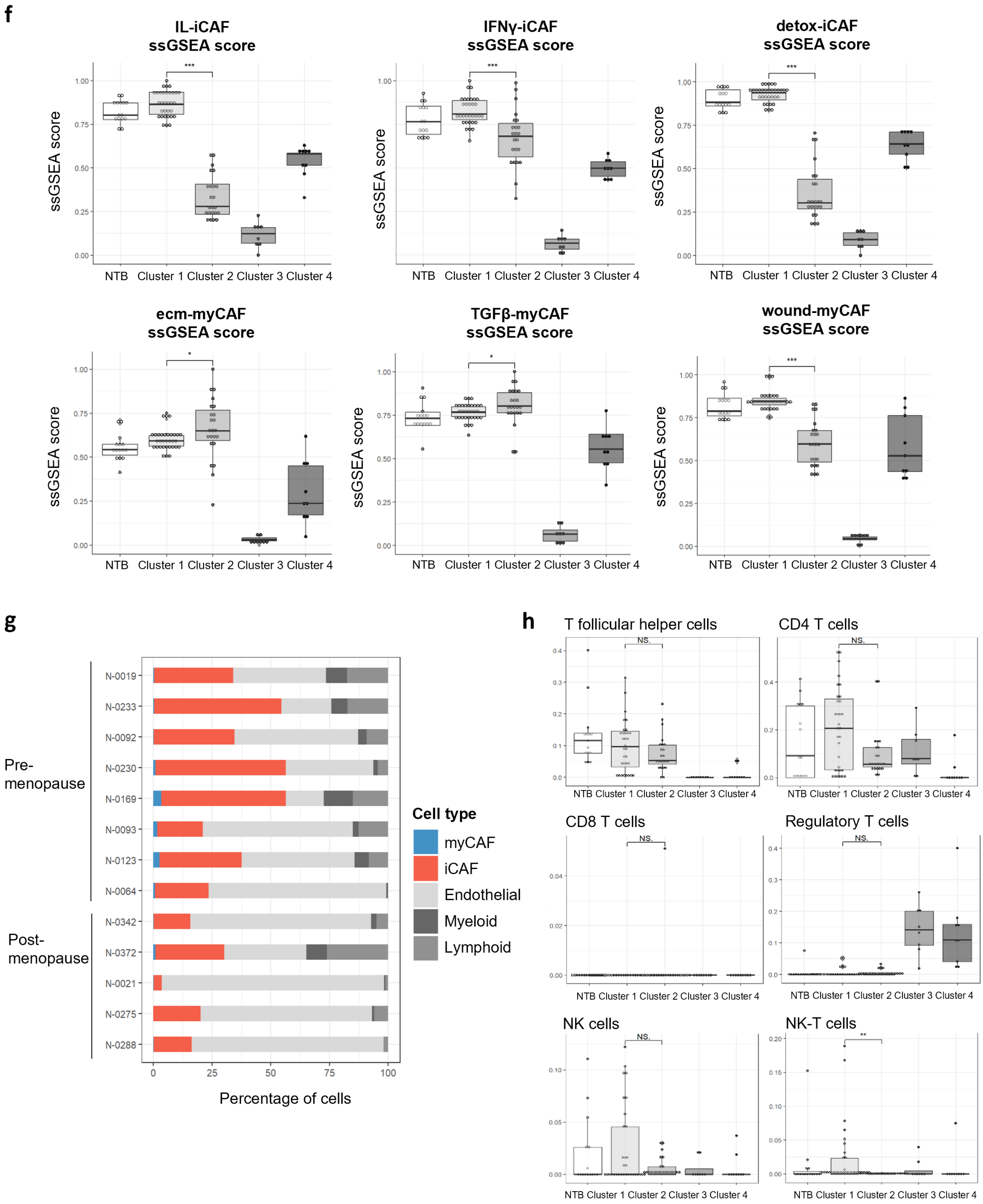

Supplementary Figure 5i-j – Lau et al.

i

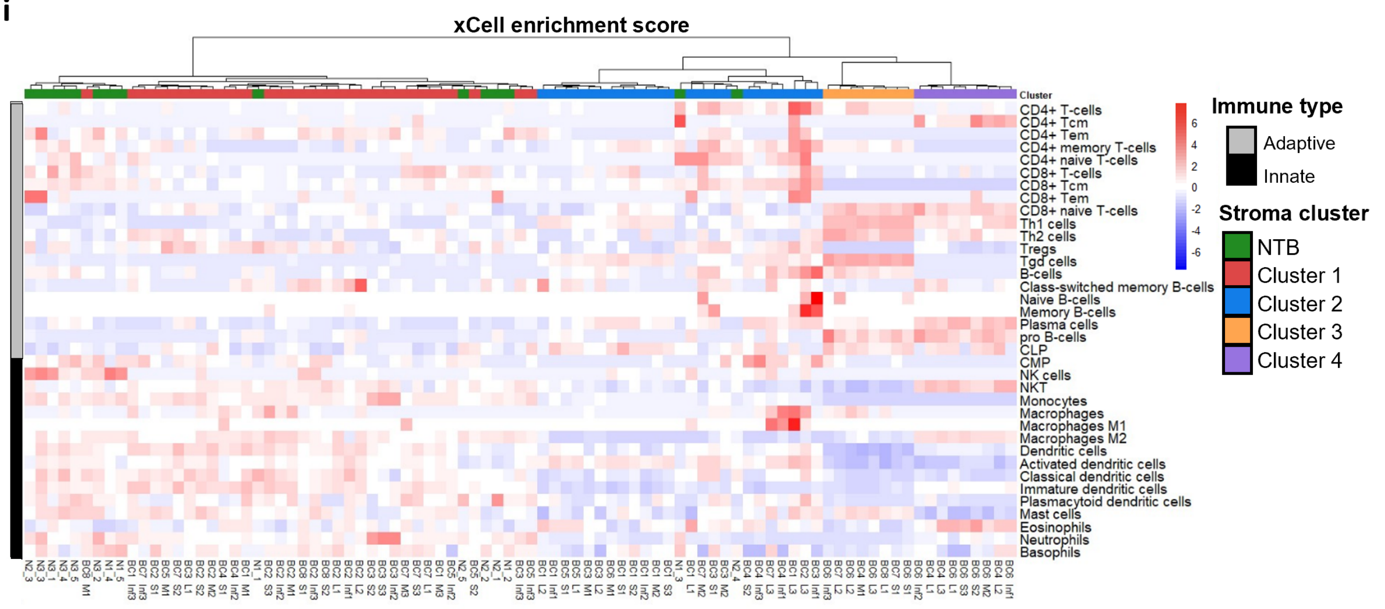

j

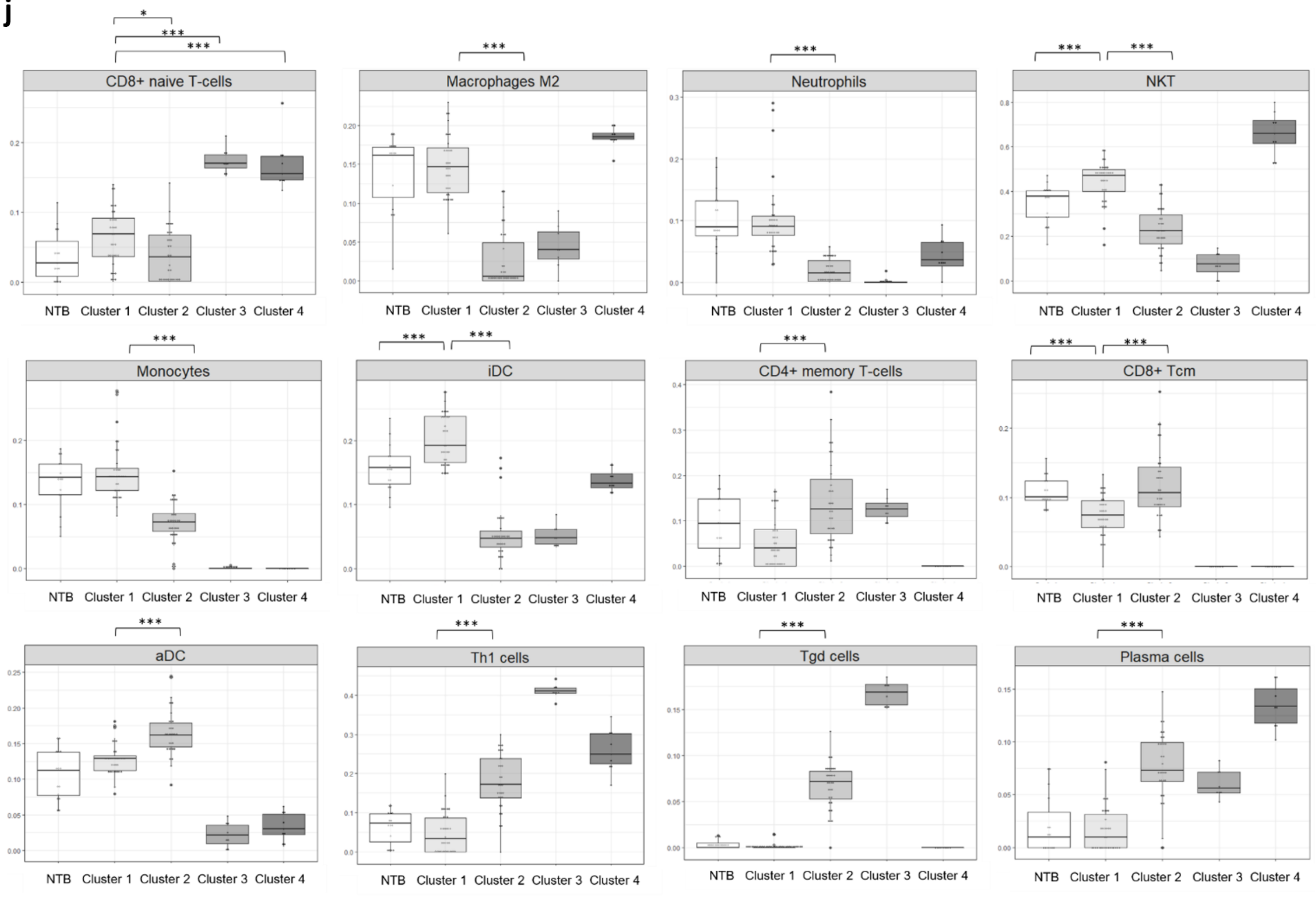

Supplementary Figure 5k-l – Lau et al.

k

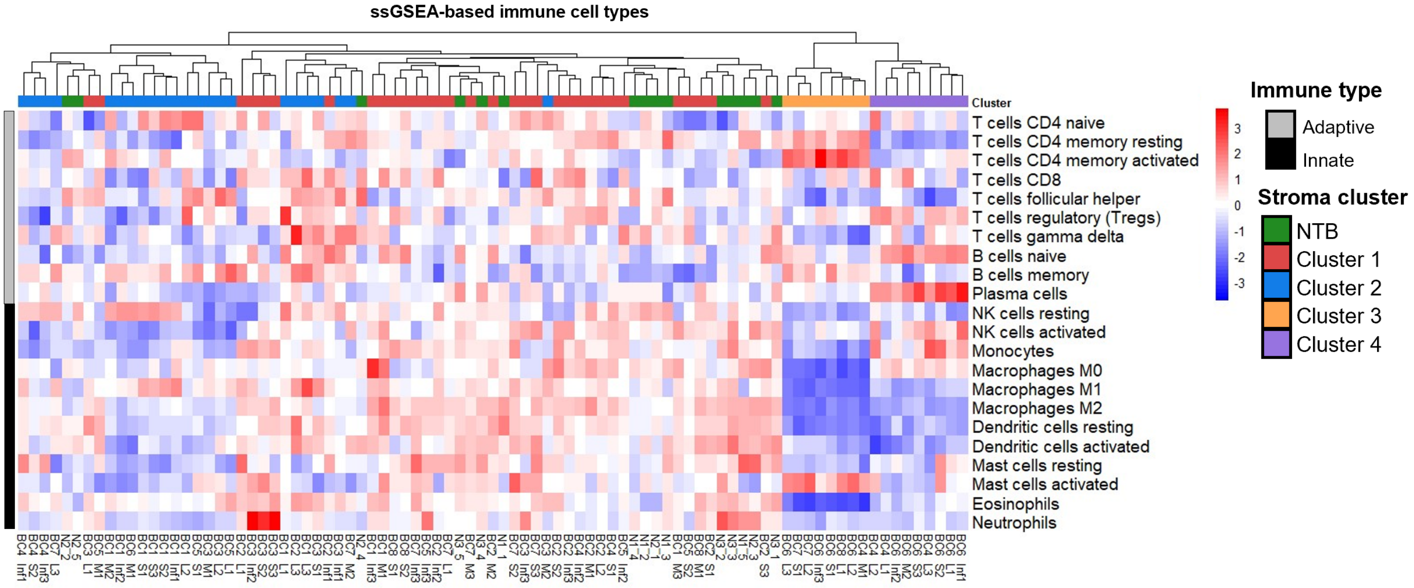

l

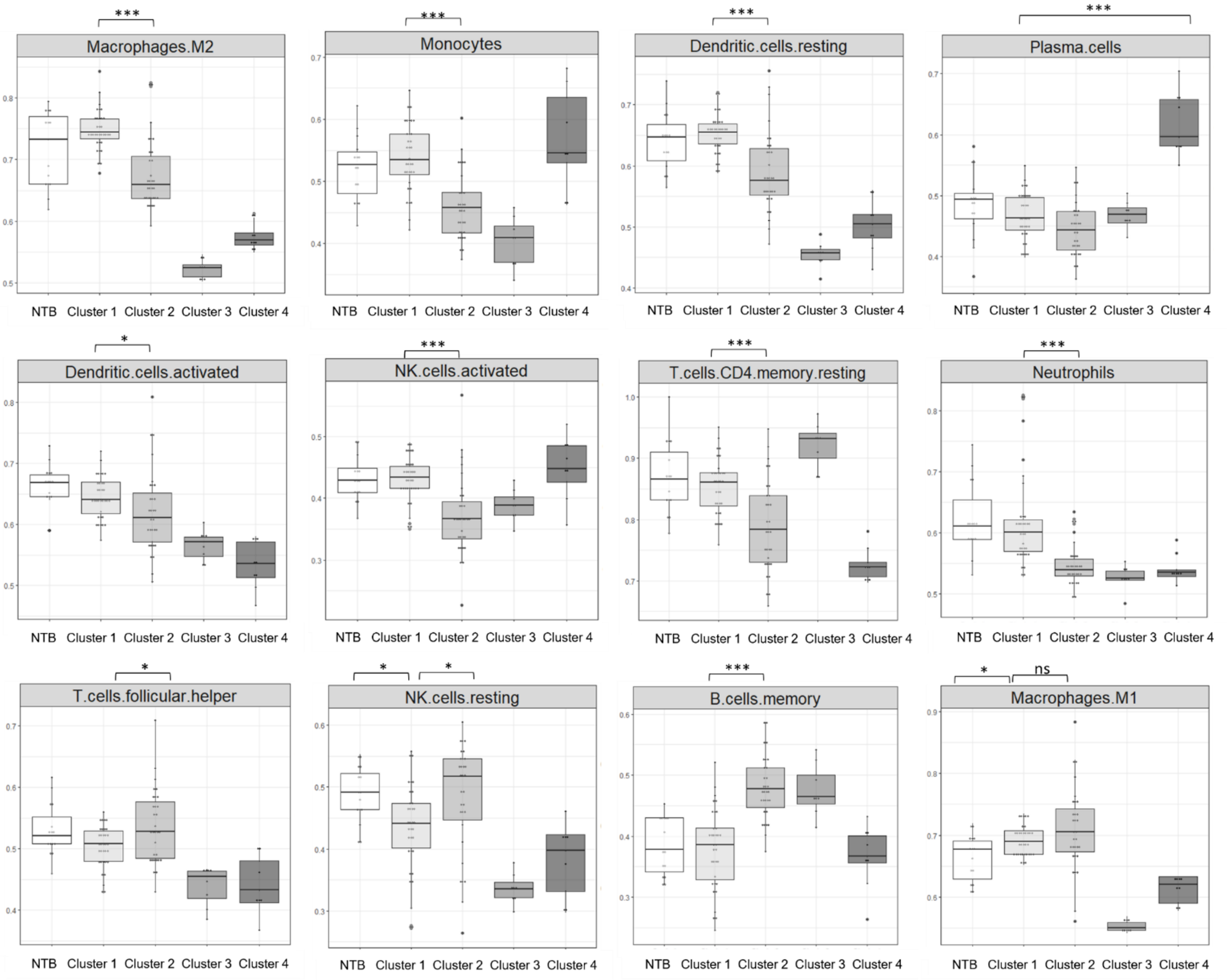

Supplementary Figure 5. Cancer-associated fibroblast subtypes and immune cell compositions vary between peri-tumoral clusters.

(a) Heatmap of myofibroblast-like CAFs (myCAFs) and inflammatory CAFs (iCAFs) signatures expressed by NTB and peri-tumoral samples (based on markers identified by scRNA-seq of human TNBC samples from Wu *et al.* (2020). **(b)** Box plots showing ssGSEA scores for iCAF and myCAF marker genes for NTB and peri-tumoral clusters. Mann-Whitney U test p-values are shown. (* $p \leq 0.05$, ** $p \leq 0.01$, *** $p \leq 0.001$). Center line represents the median, box limits show the upper and lower quartiles and whiskers show 1.5x interquartile ranges. **(c)** Estimation of myCAF and iCAF proportions in NTB and peri-tumoral samples by CIBERSORTx deconvolution (using custom signature matrix built from scRNA-seq data from Wu *et al.* (2020). **(d)** Spatial diagrams of all patients indicating cluster identity and estimated proportions of myCAFs and iCAFs in each peri-tumoral region based on CIBERSORTx deconvolution. **(e)** myCAF and iCAF proportions in TCGA tumor-adjacent normal samples divided into Cluster 1-enriched and Cluster 2-enriched groups (n=50), estimated by CIBERSORTx deconvolution using signature matrix built from scRNA-seq data from Elyada *et al*. (2019). Mann-Whitney U test p-values are shown. **(f)** ssGSEA scores for iCAF and myCAF subgroups as identified by Kieffer *et al.* (2020) in NTB and peri-tumoral samples. Mann-Whitney U test p-values are shown. **(g)** Classification of fibroblasts from scRNA-seq data of normal breast tissue (n=13) into myCAFs and iCAFs by SingleR. **(h)** Estimated proportions of immune cell types in NTB and peri-tumoral samples by CIBERSORTx deconvolution (using custom matrix built from scRNAseq-data from Wu *et al*., 2020). Mann-Whitney U test p-values are shown. **(i)** Heatmap of xCell enrichment scores of 36 immune cells in NTB and peri-tumoral samples. **(j)** Box plots of xCell enrichment scores of 12 selected immune cell types for each peri-tumoral cluster and NTB samples. Mann-Whitney U test p-values are shown. **(k)** Heatmap of ssGSEA scores of 22 immune cell types in NTB and peri-tumoral samples. **(l)** Box plots of ssGSEA scores of 12 selected immune cells for each peri-tumoral cluster and NTB samples. Mann-Whitney U test p-values are shown.
