# Supplementary material for: Adipose-enriched peri-tumoral stroma, in contrast to myofibroblast-enriched stroma, prognosticates poorer survival in breast cancers
Source: NPJ Breast Cancer. 2023 Oct 20;9:84. doi: 10.1038/s41523-023-00590-7 (PMC10589339; doi:10.1038/s41523-023-00590-7)
Supplement: Supplementary file 2 — Reporting summary [file 41523_2023_590_MOESM2_ESM.pdf]

Corresponding author(s): Kanaga Sabapathy

Last updated by author(s): Jun 13, 2023

Reporting Summary

Nature Portfolio wishes to improve the reproducibility of the work that we publish. This form provides structure for consistency and transparency in reporting. For further information on Nature Portfolio policies, see our Editorial Policies and the Editorial Policy Checklist.

Statistics

For all statistical analyses, confirm that the following items are present in the figure legend, table legend, main text, or Methods section.

n/a	Confirmed	
☐	☒	The exact sample size (*n*) for each experimental group/condition, given as a discrete number and unit of measurement
☐	☒	A statement on whether measurements were taken from distinct samples or whether the same sample was measured repeatedly
☐	☒	The statistical test(s) used AND whether they are one- or two-sided<br>*Only common tests should be described solely by name; describe more complex techniques in the Methods section.*
☐	☒	A description of all covariates tested
☐	☒	A description of any assumptions or corrections, such as tests of normality and adjustment for multiple comparisons
☐	☒	A full description of the statistical parameters including central tendency (e.g. means) or other basic estimates (e.g. regression coefficient) AND variation (e.g. standard deviation) or associated estimates of uncertainty (e.g. confidence intervals)
☐	☒	For null hypothesis testing, the test statistic (e.g. *F*, *t*, *r*) with confidence intervals, effect sizes, degrees of freedom and *P* value noted<br>*Give P values as exact values whenever suitable.*
☒	☐	For Bayesian analysis, information on the choice of priors and Markov chain Monte Carlo settings
☒	☐	For hierarchical and complex designs, identification of the appropriate level for tests and full reporting of outcomes
☒	☐	Estimates of effect sizes (e.g. Cohen's *d*, Pearson's *r*), indicating how they were calculated

Our web collection on statistics for biologists contains articles on many of the points above.

Software and code

Policy information about availability of computer code

Data collection	No software was used for data collection.
Data analysis	We used open source published software and code fully described in the manuscript with references provided. All graphs and statistical analyses were performed using R version 3.6.1 or GraphPad Prism version 8.4.3.

For manuscripts utilizing custom algorithms or software that are central to the research but not yet described in published literature, software must be made available to editors and reviewers. We strongly encourage code deposition in a community repository (e.g. GitHub). See the Nature Portfolio guidelines for submitting code & software for further information.

Data

Policy information about availability of data

All manuscripts must include a data availability statement. This statement should provide the following information, where applicable:

- Accession codes, unique identifiers, or web links for publicly available datasets
- A description of any restrictions on data availability
- For clinical datasets or third party data, please ensure that the statement adheres to our policy

Raw CEL files and processed expression data have been deposited as GEO accession number GSE211729.

Research involving human participants, their data, or biological material

Policy information about studies with human participants or human data. See also policy information about sex, gender (identity/presentation), and sexual orientation and race, ethnicity and racism.

Reporting on sex and gender	All subjects included in this study are female.
Reporting on race, ethnicity, or other socially relevant groupings	Of the 8 breast cancer patients in this study, 7 were Chinese and 1 was Malay. For the healthy subjects, 1 was Chinese, 1 was Malay and 1 was Indian.
Population characteristics	Details of the population characteristics can be found in Supplementary Table 1.
Recruitment	Patients with primary breast cancer at the National Cancer Centre Singapore (NCCS) were recruited prior to surgery and provided written informed consent for tissue harvest for the purpose of this study between 2016 and 2017.
Ethics oversight	The study was approved by the National Cancer Centre Singapore Institutional Ethics Board (approval number: CIRB2015/2722).

Note that full information on the approval of the study protocol must also be provided in the manuscript.

Field-specific reporting

Please select the one below that is the best fit for your research. If you are not sure, read the appropriate sections before making your selection.

☒ Life sciences ☐ Behavioural & social sciences ☐ Ecological, evolutionary & environmental sciences

For a reference copy of the document with all sections, see nature.com/documents/nr-reporting-summary-flat.pdf

Life sciences study design

All studies must disclose on these points even when the disclosure is negative.

Sample size	No sample size calculation was performed as this is an exploratory study. We performed comprehensive multi-regional computational analysis of 8 breast tumors and their matched peri-tumoral normal regions (39 tumor, 72 peri-tumoral) and 5 samples each from 3 patients who had undergone reduction mammoplasties or risk-reducing mastectomies (15 non-tumor bearing breast samples).
Data exclusions	Only patients with ER+/PR+/HER2- tumors were included.
Replication	NA
Randomization	NA
Blinding	NA

Reporting for specific materials, systems and methods

We require information from authors about some types of materials, experimental systems and methods used in many studies. Here, indicate whether each material, system or method listed is relevant to your study. If you are not sure if a list item applies to your research, read the appropriate section before selecting a response.

Materials & experimental systems

n/a	Involved in the study	
☒	☐	Antibodies
☒	☐	Eukaryotic cell lines
☒	☐	Palaeontology and archaeology
☒	☐	Animals and other organisms
☐	☒	Clinical data
☒	☐	Dual use research of concern
☒	☐	Plants

Methods

n/a	Involved in the study	
☒	☐	ChIP-seq
☒	☐	Flow cytometry
☒	☐	MRI-based neuroimaging

Clinical data

Policy information about clinical studies

All manuscripts should comply with the ICMJE guidelines for publication of clinical research and a completed CONSORT checklist must be included with all submissions.

Clinical trial registration	This study is not a clinical trial.
Study protocol	NA
Data collection	NA
Outcomes	NA
